# The prevalence of apathy in Lewy body dementia: A systematic review and meta‐analysis

**DOI:** 10.1002/alz.70425

**Published:** 2025-07-03

**Authors:** Jenny Jia Yu, Kai Sin Chin, Paula M. Loveland, Leonid Churilov, Samantha M. Loi, Nawaf Yassi, Rosie Watson

**Affiliations:** ^1^ Melbourne Brain Centre @ The Royal Melbourne Hospital, Department of Medicine University of Melbourne Parkville Victoria Australia; ^2^ Population Health and Immunity Division The Walter and Eliza Hall Institute of Medical Research Parkville Victoria Australia; ^3^ Neuropsychiatry Centre The Royal Melbourne Hospital Parkville Victoria Australia; ^4^ Department of Psychiatry University of Melbourne Parkville Victoria Australia

**Keywords:** apathy, behavioral and psychological symptoms of dementia, Lewy body dementia, neuropsychiatric symptoms, Parkinson's disease dementia, prodromal dementia with Lewy bodies, systematic review

## Abstract

**INTRODUCTION:**

Apathy is an important syndrome in Lewy body dementia (LBD), although reported prevalences vary. We aimed to estimate the prevalence of apathy in LBD through systematic review and meta‐analysis.

**METHODS:**

Five databases were searched for articles reporting the prevalence of apathy in LBD (dementia with Lewy bodies [DLB] and Parkinson's disease dementia [PDD]) and prodromal/mild cognitive impairment (MCI) LBD. Linear mixed model random effect meta‐analysis was performed to determine the prevalence of apathy.

**RESULTS:**

Of 7846 articles identified, 49 met inclusion criteria. The average pooled prevalence of apathy was 57% (95% confidence interval [CI] 52%–63%, *I* ^2^ = 85.6%) in DLB, 56% (95% CI 43%–70%, *I* ^2^ = 97.6%) in PDD, 46% (95% CI 32%–61%, *I* ^2^ = 90.6%) in Lewy body‐MCI, and 38% (95% CI 25%–50%, *I* ^2^ = 88.1%) in Parkinson's disease‐MCI.

**DISCUSSION:**

Apathy affects more than half of individuals with LBD. The high prevalence in prodromal DLB supports the notion that apathy may assist in more timely and accurate diagnosis of DLB.

**Highlights:**

- Reported apathy prevalence in Lewy body dementia (LBD) varies widely in the existing literature.
- The pooled prevalence of apathy was > 50% in LBD and > 40% in prodromal disease stages.
- Apathy may have utility in earlier, more accurate diagnosis of dementia with Lewy bodies.

## 1 BACKGROUND

Dementia with Lewy bodies (DLB) and Parkinson's disease dementia (PDD), collectively known as the Lewy body dementias (LBDs), are characterized clinically by a complex combination of progressive cognitive, motor, and neuropsychiatric manifestations. While visual hallucinations are characteristic of the conditions (being a core clinical feature of DLB), it is well recognized that other neuropsychiatric symptoms are prevalent in LBD. In particular, apathy, a neuropsychiatric syndrome of persistent diminished initiative, interest, or emotional expression or responsiveness,^1^ is often seen in patients with LBD, and its occurrence may have various implications depending on the stage of disease.

There is emerging evidence that apathy plays an important role in the initial presentation of LBD and may signal subclinical or future cognitive impairment. For example, an association between apathy and poorer cognition in Parkinson's disease (PD) without dementia has been previously demonstrated.^2^ Similarly, prodromal DLB can present as an apathy syndrome, prior to the onset of cognitive decline.^3^ A recent study revealed the presence of apathy (or anxiety or depression), alongside at least one core clinical feature of DLB,^4^ distinguished prodromal DLB from healthy controls with high accuracy (area under the curve [AUC] of 0.815).^5^

Apathy is also an important intervention target in established LBD, where it compounds the morbidity caused by cognitive decline and non‐cognitive features of the disease. Apathy in DLB is associated with faster global cognitive decline and shorter time to residential care admission compared to apathy in AD.^6^ While the prevalence of apathy does not seem to increase with disease progression in DLB,^7^, ^8^ apathy severity in PDD worsens as cognition declines^9^ and heightens the functional impairment caused by cognitive and motor symptoms.

Despite its significance, apathy in LBD has not been well defined in terms of prevalence or clinical implications. Early studies in LBD often focused on the overall burden of neuropsychiatric symptoms^7^, ^8^, ^10^ or psychotic symptoms in DLB,^11^ rather than apathy. Neuropsychiatric symptoms associated with PD have also been widely studied, but most studies have excluded individuals with dementia^12^, ^13^, ^14^ or did not report PDD as separate from PD.^15^ The prevalence of apathy in LBD varies widely in the existing literature. Some of this variability may reflect small study sample sizes, the use of different apathy assessment tools, and the challenges in differentiating comorbid and overlapping conditions such as depression and anxiety. Leung et al.’s recent systematic review and meta‐analysis of affective symptoms (depression, anxiety, and apathy) in various dementia types included only one DLB study.^16^ A 2015 systematic review and meta‐analysis of apathy in PD, which included a cohort with dementia, did not report the dementia subgroup's apathy prevalence separately.^17^ Additionally, it defined dementia in PD using the now superseded Diagnostic and Statistical Manual (DSM) criteria or a low score on cognitive testing, rather than the current Movement Disorder Society Task Force criteria.^18^ Therefore, we aimed to conduct a systematic review and meta‐analysis to estimate the prevalence of apathy in LBD and prodromal LBD. As apathy is known to be associated with poor clinical outcomes, we also aimed to explore how clinical correlates of apathy were investigated in the articles we included for our systematic review.

**RESEARCH IN CONTEXT**

1. **Systematic review**: The authors reviewed five databases for relevant articles. The reported prevalence of apathy in Lewy body dementia (LBD), which includes dementia with Lewy bodies (DLB) and Parkinson's disease dementia (PDD), varied widely. Many articles suggested that apathy was associated with poor clinical outcomes.
2. **Interpretation**: Apathy affects at least 1 in 2 people with LBD throughout the disease continuum. The high prevalence of apathy in prodromal DLB suggests that apathy may assist in more timely and accurate diagnosis of DLB. Methods used to analyze clinical correlates of apathy are highly heterogenous. There was a lack of longitudinal data on clinical correlates, which is needed to better characterize apathy longitudinally and understand its prognostic significance.
3. **Future directions**: Future research should investigate the impact of incorporating apathy as part of diagnostic criteria for DLB, especially prodromal disease.

## 2 METHODS

We reported our systematic review and meta‐analysis according to the Preferred Reporting Items for Systematic Reviews and Meta‐Analyses (PRISMA) statement. The protocol was registered prospectively with PROSERO: CRD42021292044.

### 2.1 Search strategy

Five databases (Embase, Medline, PsycInfo, Web of Science, and CINAHL) were systematically searched for relevant articles. Search terms used included: “Lewy body,” “Parkinson's disease dementia,” “mild cognitive impairment (MCI),” “neuropsychiatric symptoms,” “apathy,” and their synonyms. Full search strategies are detailed in Appendix A in supporting information. The last search was performed on August 26, 2024.

### 2.2 Eligibility criteria and data extraction

Two authors (J.J.Y. working with K.S.C. or P.M.L.) independently screened the titles and abstracts. A third independent reviewer (R.W.) was available if a decision for inclusion of an article could not be made by the primary two reviewers. The same authors completed the full text review independently.

Articles for data extraction were selected using the following inclusion criteria:

1. Articles reporting on a clinically or neuropathologically diagnosed DLB or PDD or prodromal/MCI DLB/PDD cohort (based on current consensus diagnostic criteria^3^, ^4^, ^18^, ^19^ or their previous versions) of humans of any age.
2. Articles reporting a minimum of the following baseline participant characteristics: age, sex, and at least one standardized measure of dementia severity (for example, the Clinical Dementia Rating [CDR] or the Mini‐Mental State Examination [MMSE]).
3. Articles reporting the prevalence of apathy, diagnosed by a standardized tool.
4. Cohorts of at least 10 individuals in each relevant diagnostic group.

Given the typically small sample size of LBD studies, a minimum sample size of 10 was chosen to maximize the inclusivity of study selection and allow a meaningful representation of the available literature.

### 2.3 Data collection

Data extracted included: study design, sample size, demographics, diagnosis, diagnostic criteria used, measure of cognitive impairment (CDR, MMSE, etc.), measures of motor symptoms (e.g., Unified Parkison's Disease Rating Scale Part III [UPDRS III] or the Hoehn & Yahr [H&Y] scale), relevant medications, measure of neuropsychiatric symptom burden (e.g., total Neuropsychiatric Inventory [NPI] score), proportion of participants with apathy, method of apathy diagnosis, and whether clinical correlates (cognition, function, residential age of admission, mortality, and caregiver outcomes) were assessed. Diagnostic groups of interest were defined as DLB, PDD, LBD (which included neuropathologically diagnosed LBD groups as well as combined DLB/PDD cohorts), Lewy body mild cognitive impairment (LB‐MCI, sometimes referred to as prodromal DLB), and PD with MCI (PD‐MCI).

### 2.4 Study quality assessment

Study quality assessment was performed by two independent reviewers (J.J.Y. and K.S.C.) using the Joanna Briggs Institute Checklist for Prevalence Studies.^20^

### 2.5 Statistical analysis

Statistical analysis was completed using Stata version 18 software (StataCorp LLC). Average pooled prevalence was estimated using generalized linear mixed model random‐effect meta‐analysis, with exact binomial 95% Clopper–Pearson confidence intervals (CIs) across the diagnostic groups: DLB, PDD, LBD, LB‐MCI, and PD‐MCI. Heterogeneity of estimates (the proportion of the variation in observed effects that is due to variation in true effects)^21^ was assessed using the *I* ^2^ statistic.^22^ Certain participant cohorts were used by more than one article included in our analysis. These “overlapping cohorts” were identified by reviewing the recruitment processes of articles with the same authors. Studies which recruited from the same hospitals or clinics during overlapping time periods or studies which referred to the same named cohort as another were deemed to have overlapping cohorts. To account for these “overlapping cohorts,” we performed a sensitivity analysis in which the articles reporting the smaller sample sizes were removed and analysis performed on the remaining studies. Given large variation in study sample size was a potential reason for heterogeneity of reported apathy prevalences, post hoc sensitivity analysis was also performed in articles with sample sizes > 50. Sensitivity analysis was also performed in articles which used NPI for the same reason.

## 3 RESULTS

### 3.1 Study characteristics

**FIGURE 1:**
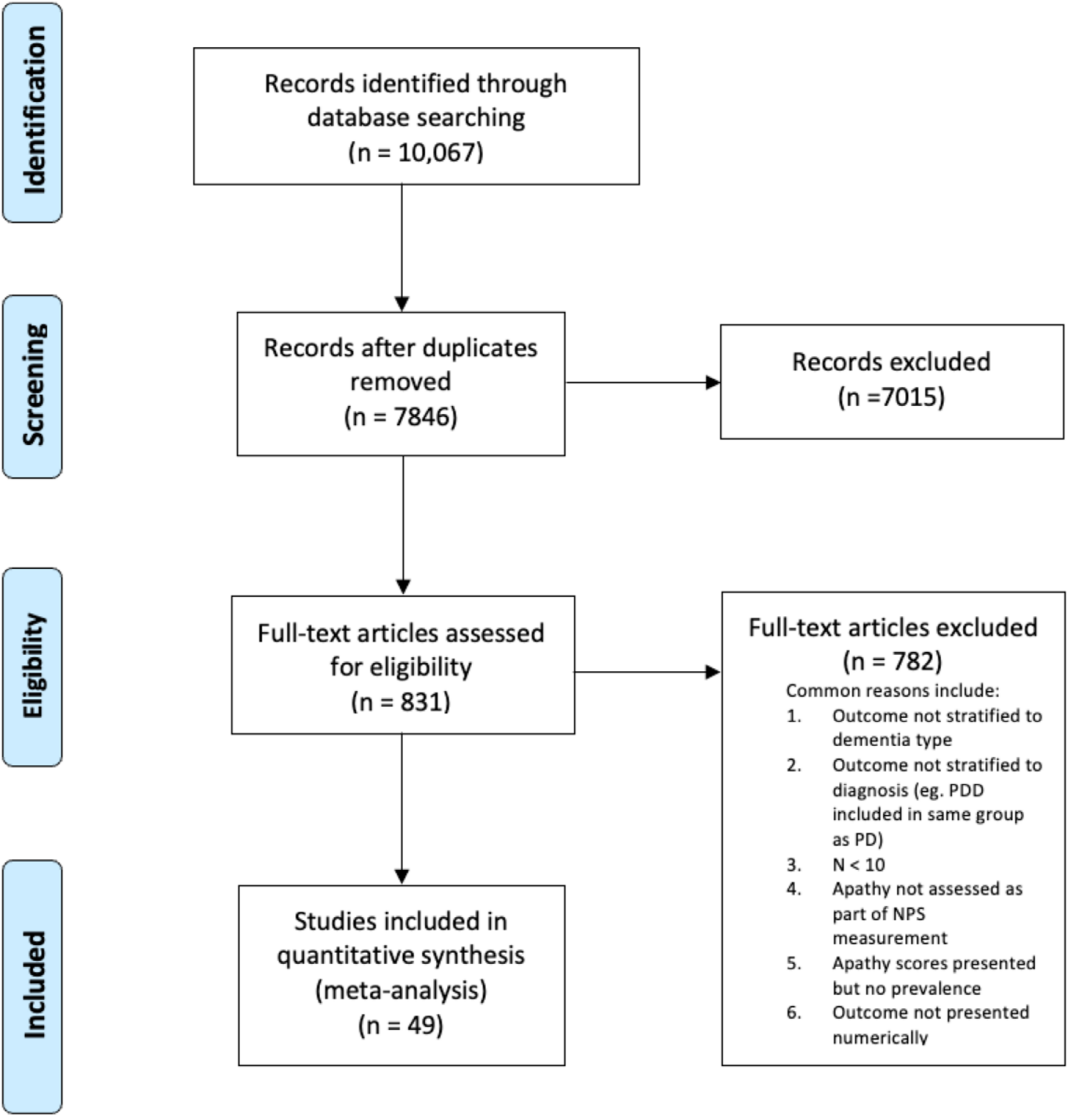
PRISMA flow diagram. NPS, neuropsychiatric symptoms; PD, Parkinson's disease; PRISMA, Preferred Reporting Items for Systematic Reviews and Meta‐Analyses

**FIGURE 2:**
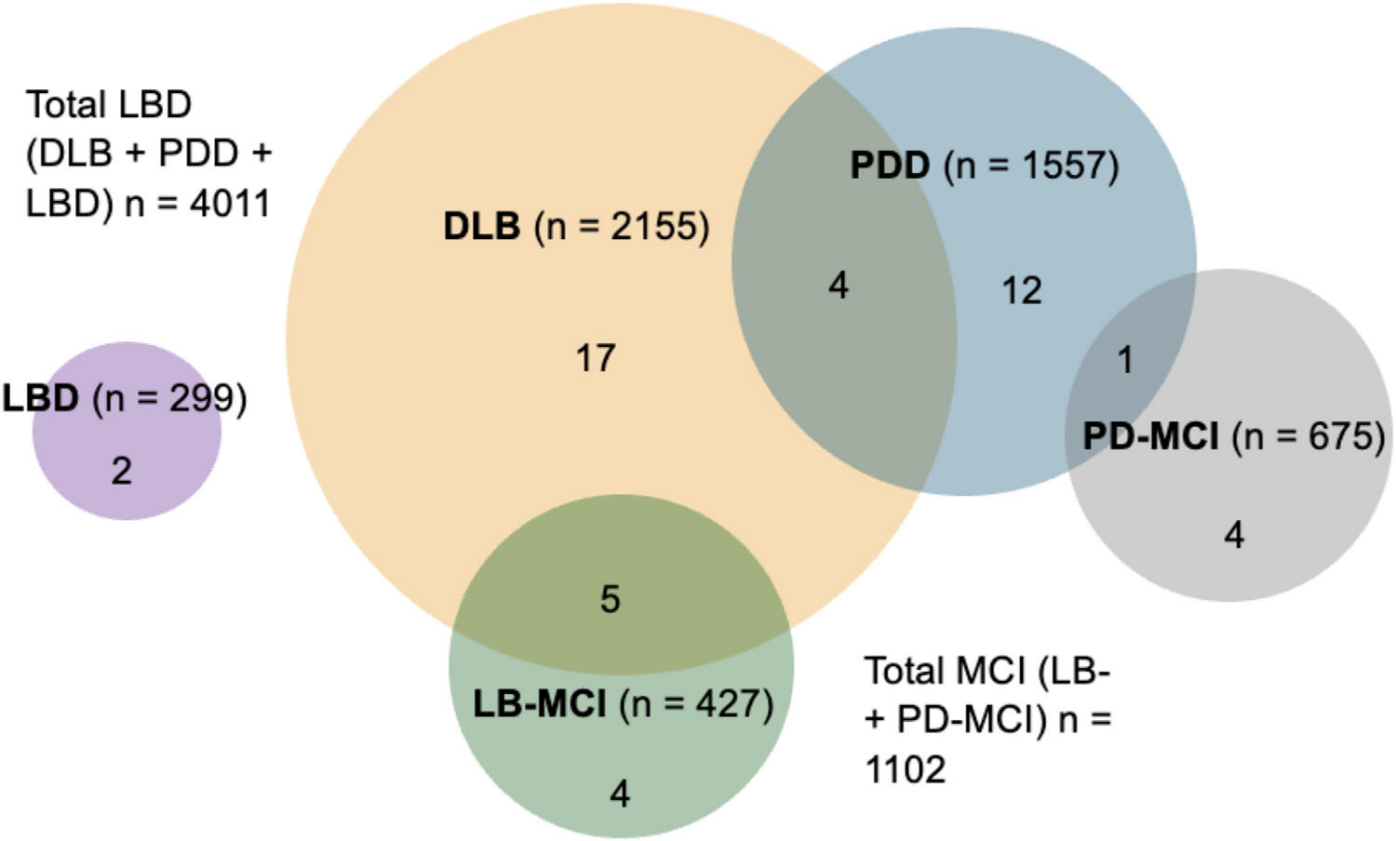
Number of articles reporting each diagnostic group of interest (total participants in each group). DLB, dementia with Lewy bodies; LBD, Lewy body dementia; LB‐MCI, Lewy body mild cognitive impairment; PDD, Parkinson's disease dementia; PD‐MCI, Parkinson's disease with mild cognitive impairment

After screening 7846 abstracts, 49 articles (with a total of 5113 participants) were included in this review (Figure 1). Reasons for excluding articles are summarized in Figure 1. Twenty‐six articles reported on DLB cohorts, seventeen on PDD, nine on LB‐MCI, five on PD‐MCI, and two on LBD (in which DLB and PDD participants were combined into one group). Ten of the 49 articles reported on multiple diagnostic groups (Figure 2).

Table 1 summarizes the study characteristics of included articles. This review included 2155 DLB, 1557 PDD, 427 LB‐MCI, and 675 PD‐MCI participants (Figure 2). Two articles combined DLB and PDD participants into an LBD cohort, with a total of 299 participants. The median sample size was 51 (range 12 to 537). Thirty‐seven articles examined neuropsychiatric symptoms as their primary aim; of these, five articles focused specifically on apathy. The remaining articles reported apathy prevalence as part of baseline participant characteristics. All except six articles used the NPI to assess apathy. Other apathy assessment tools included the Apathy Evaluation Scale (AES), Lille Apathy Rating Scale (LARS), Blessed Dementia Scale, the Starkstein Apathy Scale (SAS), and the Lewy Body Dementia Association Comprehensive Checklist. Eighteen of the included articles reported at least one clinical correlate of apathy.

**TABLE 1 alz70425-tbl-0001:** Characteristics of included studies.

								Clinical correlates of apathy						
Study	Diagnostic groups/ diagnostic criteria	Inclusion/exclusion criteria	N (total, males %)	Age, mean (SD)	Degree of cognitive impairment, mean (SD) or median [IQR]	Apathy scale used (cut‐off for apathy diagnosis)	Apathy prevalence	Cognition	Function	Motor impairment	Quality of life	Residential care admission	Mortality	Caregiver burden
DLB (26 studies)														
Mori 2006^23^ Japan	**DLB** McKeith 1996	Inclusion: CDR 0.5, 1, or 2; MMSE ≥ 10, presence of caregivers for ≥ 5 days per week Exclusion: Hoehn & Yahr IV or above	12, 50%	78.7 (5.1)	MMSE 19.3 (4.9) CDR‐GS 0.5: 5 1: 6 2: 1	NPI (cut‐off score not specified)	**75%**							
McKeith 2006^24^ UK	**DLB**, AD McKeith 1996	None specified	41, 53.7%	76.4 (6.8)	MMSE 16.2 (5.3)	NPI (cut‐off score not specified)	**53.7%**							
Galvin 2007^25^ USA	**DLB**, AD, HC McKeith 2005 (neuropathological)	Inclusion: cases that came to autopsy	128, 50.8%	80.8 (10.4)	CDR‐GS 0.79 (0.45) CDR‐SOB 4.5 (3)	Blessed Dementia Scale (“presence” of apathy)	**47.1%**							
Caputo 2008^7^ Italy	**DLB**, VaD, AD McKeith 1996 (clinical)	Exclusion: dementia due to other general medical conditions, a history of alcohol or substance abuse or dependence, uncertain dementia, delirium, and psychiatric disorders preceding the onset of dementia	100, 49%	77.8 (7.5)	MMSE 14 (7.4) CDR‐GS 1: 21 2: 47 3: 32	NPI (apathy subscale frequency × severity ≥ 4)	**60%**							
Ricci 2009^26^ Italy	**DLB**, AD McKeith 2005	Inclusion: Hamilton Depression Scale < 17, MMSE > 14 Exclusion: taking neuroleptics	16, 62.5%	74.7 (4.3)	MMSE 20.7 (4.5) CDR‐SOB 1.5 (0.5)	NPI (cut‐off score not specified)	**100%**	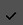						
Johnson 2011^10^ USA	**DLB**, PDD, AD+DLB, AD, AD+VaD, VaD McKeith 2005	Inclusion: CDR ≥ 1, NPI‐Q data available, ≥ 60 years	151, 74.2%	69.4 (8.7)	CDR‐GS 1: n, 91, 2: n, 42, 3: n, 18	NPI (cut‐off score not specified)	**73%**	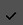						
Bjoerke‐Bertheussen 2012^8^ Norway	**DLB**, AD McKeith 1996	Inclusion: new diagnosis of dementia Exclusion: acute delirium or confusion	57, 56.1%	76.6 (7.9)	MMSE 23.1 (3.4)	NPI (apathy subscale frequency × severity ≥ 4)	**56.1%**							
Marra 2012^27^ Italy	**DLB**, AD McKeith 2005	None specified	18, 61.1%	73 (4.6)	MMSE 18.9 (5.4)	NPI (cut‐off score not specified)	**83.3%**							
Yoshida 2015^28^ Japan	**DLB**, HC McKeith 2005	Inclusion: living with at least one caregiver, had visual hallucinations Exclusion: left‐handed, taking acetylcholinesterase inhibitors, past histories of psychiatric diseases, obvious cerebrovascular lesions in MRI or CT	27, 51.9%	77.5 (5.5)	MMSE 18.8 (5.5)	NPI (apathy subscale frequency × severity > 0)	**51.9%**							
*Chiu 2016* ^29^ *Taiwan*	**DLB**, PDD, AD McKeith 2007	None specified	250, 53.6%	78 (6.6)	MMSE 15.1 (7) CDR‐SOB 8.5 (3.8)	NPI (cut‐off score not specified)	**61.6%**							
Perri 2016^30^ Italy	**DLB**, AD, bvFTD, sub‐cortical isch‐aemic VaD McKeith 2005	Inclusion: mild to moderate dementia (CDR GS < 3) Exclusion: using anticholinesterase or neuroleptic therapy	21, 61.9%	72.6 (6.4)	MMSE 20.8 (3.4) CDR‐GS 0.8 (0.6)	NPI (cut‐off score not specified)	**61.9%**							
Siepel 2016^31^ Norway	**DLB** McKeith 2005	None specified	35, 54.3%	74.3 (7.4)	MMSE 25 (5)	Starkstein Apathy Scale (score > 13)	**57.1%**							
*Donaghy 2017* ^32^ *England*	**DLB**, LB‐MCI, AD, AD‐MCI, HC McKeith 2005	Inclusion: MMSE > 11 Exclusion: major concurrent psychiatric illness; severe physical illness; other significant neurological illness; current treatment with any investigational agent.	36, 86.1%	75.7 (0.3)	MMSE 21.5 (4.6)	The Lewy Body Dementia Association Comprehensive Checklist	**47%**							
Breitve 2018^6^ Norway	**DLB**, AD McKeith 1996	Inclusion: new diagnosis of dementia Exclusion: acute delirium or confusion	76, 50%	no apathy: 77.3 (5.9) apathy: 5.9 (8.2)	CDR‐GS no apathy: 0.9 (0.5) apathy 1.1 (0.4)	NPI (apathy subscale frequency × severity ≥ 4)	**48.7%**	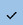				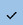	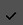	
Tsunoda 2018^33^ Japan	**DLB** McKeith 2005	Exclusion of serious psychiatric diseases such as schizophrenia, major depression, or substance abuse before the onset of dementia; evidence of focal brain lesions on MRI; no reliable informant; and unable to provide informed consent	124, 43.5%	78.3 (5.6)	MMSE 19.1 (5.7)	NPI (cut‐off score not specified)	**58.9%**							
VanDeBeek 2019^34^ Netherlands	**DLB**, AD, HC McKeith 2005	Inclusion: quality of life data available	29, 93.1%	69 (6.3)	MMSE 25 [22– 27]	NPI (apathy subscale frequency × severity > 0)	**79%**				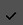			
*Liu 2020* ^35^ *China*	**DLB**, LB‐MCI, AD, AD‐MCI McKeith 2017	Exclusion: mixed dementia aetiology (e.g., severe hippocampal atrophy in LB‐MCI), major concurrent psychiatric illness or a history of other significant neurological illness, including stroke or alcohol/substance abuse history, lack of caregiver	97, 46.4%	75 [70–90]	MMSE 14 [8– 18] MoCA 8 [5– 12]	NPI (cut‐off score not specified)	**52.6%**							
Yoo 2020^36^ South Korea	**DLB** McKeith 2017	Inclusion: dopaminergic depletion on 18F‐FP‐CIT Exclusion: severe white matter hyperintensities, multiple lacunes in the basal ganglia, or hydrocephalus on MRI; neurologic, psychiatric, or metabolic illnesses; and history of antiacetylcholine esterase inhibitor or antipsychotic agents	51, 51%	Amyloid negative: 79.38 (4.92) Amyloid positive: 74.82 (7.26)	MMSE amyloid negative: 20.95 (3.75); amyloid positive: 18.65 (5.36) CDR‐SOB Amyloid negative: 5.42 (3.42); amyloid positive: 6.14 (4.39)	NPI (cut‐off score not specified)	**66.7%**							
Jaramillo‐Jimenez 2021^37^ Norway	**DLB** and AD McKeith 1996	Inclusion: mild dementia (MMSE ≥ 20 OR CDR global score, 1); subjects with baseline high‐resolution structural MRI Exclusion: delirium personal history of bipolar or psychotic disorder, terminal illness recently diagnosed major somatic illness	34, 64.7%	76.3 (7.01)	MMSE 24 (5)	NPI (apathy subscale frequency × severity ≥ 4)	**44.1%**							
*Galvin 2021* ^38^ *USA*	**DLB**, LB‐MCI, AD, AD‐MCI McKeith 2010	None specified	110, 72.7%	77.7 (7.6)	CDR‐GS 1.6 (0.8) CDR‐SOB 8.7 (4.8)	NPI (cut‐off score not specified)	**71.8%**							
*VanDeBeek 2021* ^39^ *Netherlands*	**DLB**, LB‐MCI McKeith 2005, 2017 and 2020	Inclusion: MMSE > 19 or CDR, 1, available baseline high resolution MRI Exclusion: physical life threatening conditions, nursing home	73, 89% (70 with apathy data)	70 (5)	MMSE 24 (3)	NPI (apathy subscale frequency × severity > 0)	**64%**		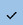		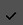			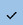
*Gan 2022* ^40^ *China*	**DLB**, LB‐MCI, HC McKeith 2017	Exclusion: Disagreement in diagnosis; other neurological disease; currently using orexin receptor antagonists; history of mental disorders and illicit drug abuse; acute or chronic liver and kidney dysfunction, malignant tumors, or other serious underlying disease	53, 34%	73.04 (7.17)	MMSE 15.11 (5.54) MoCA 11.4 (4.56) CDR‐GS 1.72 (0.72)	NPI (cut‐off score not specified)	**43.4%**	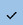						
*Gan 2022* ^41^ *China*	**DLB**, PDD McKeith 2017	Inclusion: CDR 1 or 2, > 50 years of age Exclusion: see Gan 2022 above	92, 54.3%	71 [66–78]	MMSE 18 [14–24] MoCA 12 [8–18.75] CDR‐GS 1 [1– 2]	NPI (apathy subscale frequency × severity > 0)	**45.7%**							
*Schwertner 2022* ^42^ *Sweden*	**DLB**, PDD, AD, VaD, mixed, FTD, unsp‐ecified McKeith 2017	Inclusion: individuals who were registered for Behavioral and psychological symptoms of dementia registry	236, 50%	79.6 (6.6)	MMSE 21.3 (4.9)	NPI (cut‐off score not specified)	**28.9%**							
Sim 2022^43^ Singapore	**DLB** (early and late onset), AD Neuro‐pathol‐ogical	Inclusion: Braak stage IV and below, who either had Lewy body pathology at autopsy or were given a pathological diagnosis of DLB pathology	Early onset: 32, 71.9%; late onset: 147, 70.1%	Early onset: 57.9 (3.2); late onset 78.4 (5.9)	Early onset MMSE: 22.8 (6.1); late onset MMSE: 20.6 (7.1)	NPI (cut‐off score not specified)	**57.5%** (early onset: 71.9%; late onset: 54.4%)							
Wyman‐Chick 2022^44^ USA	**DLB**, HC McKeith (version unclear)	Inclusion: primary etiology of DLB for 2 consecutive years, absence of PD, and an indication that AD was not the primary cause of observed cognitive impairment	116, 82.8%	75.6 (7.7)	MMSE (n, 67) 23.6 (4.8) MoCA (n, 39) 18.6 (5.2)	NPI (cut‐off score not specified)	**51.7%**							
PDD (17 studies)														
Aarsland 2001^45^ Norway (PDD data), USA (AD data)	**PDD**, AD DSM III‐R	Inclusion: cognitive impairment onset after motor symptoms Exclusion: previous antipsychotic use	42, 40.5%	78.2 (6.8)	MMSE 18.8 (5.7)	NPI (apathy subscale frequency × severity > 0)	**23.8%**			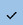				
Aarsland 2007^46^ Austria, Belgium, Canada, France, Germany, Italy, The Netherlands, Norway, Portugal, Spain, Turkey and the UK	**PDD** DSM IV	Inclusion: MMSE 10–24, onset of dementia at least 2 years after diagnosis of PD; suitable caregiver Exclusion: other neurodegenerative disorder; a major depressive episode; active, uncontrolled seizure disorder; hypersensitivity to drugs similar to rivastigmine in structure or pharmacological action	537, 65%	72.6 (6.61)	MMSE 19.3 (3.90)	NPI (apathy subscale frequency × severity ≥ 4)	**54.2%**			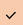				
Dujardin 2007^47^ France	**PDD**, PD and HC DSM IV	None specified	39, 43.6%	68.6 (8.91)	Mattis dementia rating scale 118.67 (7.84)	LARS (score > 15)	**56.4%**	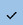		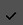				
*Johnson 2011* ^10^ *USA*	DLB, **PDD**, AD+DLB, AD, AD+VaD, VaD Litvan 2003	Inclusion: CDR ≥ 1, NPI‐Q data available, ≥ 60 years	74, 82.4%	69.5 (8.7)	CDR‐GS categories 1: n, 52; 2: n, 13; 3: n, 9	NPI (cut‐off score not specified)	**52.7%**	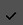						
Lee 2012^48^ Taiwan	**PDD** MDS Task Force 2007	Exclusion: developed dementia within 1 year after PD onset or who had other diseases that could impair cognition	127, 59.8%	77 (6.3)	MMSE 17 (6.5)	NPI (apathy subscale frequency × severity > 0)	**52%**	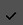						
*Leroi 2012* ^49^ *UK*	**PDD**, PD‐MCI, PD MDS Task Force 2007	None specified	25, 52%	75.58 (7.47)	MMSE 19.36 (5.96)	NPI (apathy subscale frequency × severity > 0)	**52%**	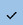						
Tsai 2014 Taiwan	**PDD** MDS Task Force 2007	Inclusion: CNS medications stable for at least 28 days and kept unchanged during study Exclusion: vascular dementia, systemic illness or drug intoxication, that could be attributed to the generation of dementia; H&Y stage IV or more	30, 63.3%	Treat‐ment 76.3 (5.3), placebo 77.3 (6.6)	Treatment MMSE: 14.9 (6.4); placebo MMSE: 19.7 (7) Cognitive abilities screening instrument Treatment: 50.9 (23.8), placebo: 63.9 (23.9)	NPI (cut‐off score not specified)	**66.7%**							
Oh 2015^50^ South Korea	**PDD** MDS Task Force 2007	Exclusion: previous stroke or other neurological and psychiatric disorders, atypical PD, secondary Parkinsonism, secondary causes of dementia	48, 58.3%	74.7 (6.6)	MMSE 19.8 (4) CDR‐GS 1.1 (0.7)	NPI (cut‐off score not specified)	**70.8%**							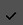
Oh 2015^51^ South Korea	**PDD** MDS Task Force 2007	Inclusion: stable Parkinson's medications 1 month prior to beginning of trial Exclusion: history of stroke, other neurological and psychiatric disorders, atypical PD, secondary Parkinsonism, secondary causes of dementia, undergoing other clinical research or were taking the study medication for other metabolic disorders, or were pregnant	23, 47.8%	74.7 (5.9)	MMSE 19.1 (4.2) CDR‐GS 1.1 (0.6)	NPI (cut‐off score not specified)	**56.5%**							
Camargo 2016^52^ Brazil	**PDD** MDS Task Force 2012	Exclusion: advanced clinical conditions of the disease; psychotic symptoms; other dementia type	39, 64.1%	70 (11)	SCOPA‐COG 10.82 (6.03)	AES‐I Brazilian (score > 14)	**97.4%**							
*Chiu 2016* ^29^ *Taiwan*	DLB, **PDD**, AD MDS Task Force 2012	None specified	125, 42.4%	77 (7)	MMSE (16.7 (7.4) CDR‐SOB 8.1 (4)	NPI (cut‐off score not specified)	**55.2%**							
Xing 2016^53^ China	**PDD**, PD, HC MDS Task Force 2007	None specified	38, 55.2%	72.7 (8)	MMSE 18.3 (4.9)	NPI (cut‐off score not specified)	**44.7%**							
Moretti 2017^54^ Italy	**PDD** MDS Task Force 2007	Exclusion: history of stroke or brain hemorrhage, psychiatric disorders, atypical PD, secondary Parkinsonism	48, 58.3%	70.4 (2.3)	MMSE 23.7 (0.6)	NPI (cut‐off score not specified)	**77.1%**							
Camargo 2018^9^ Brazil	**PDD,** PD MDS Task Force 2012	Exclusion: advanced stage PD, severe psychotic symptoms, and another dementia	40, 56.5%	70.1 (10.8)	SCOPA‐COG 11.25 (6.05)	AES (score > 14)	**97.5%**	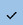						
Gryc 2020^55^ USA	**PDD**, PD MDS Task Force 2007	Exclusion: unknown cognitive diagnosis	108, 84.3%	73.4 (8)	MoCA 17.9 (4.6)	NPI (apathy subscale frequency × severity > 0)	**44.4%**	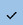						
*Gan 2022* ^41^ *China*	DLB, **PDD** MDS Task Force 2007	Inclusion: CDR 1 or 2, > 50 years of age Exclusion: see Gan 2022 above	93, 44.1%	67 [62–63]	MMSE 24 [20–26] MoCA 17 [13–20] CDR‐GS 1 [1–1]	NPI (apathy subscale frequency × severity > 0)	**16.1%**							
*Schwertner 2022* ^42^ Sweden	DLB, **PDD**, AD, VaD, mixed, FTD, un‐specified MDS Task Force 2007	Inclusion: individuals who were registered for BPSD registry	122, 61.5%	78 (6.9)	MMSE 20.8 (4.3)	NPI (cut‐off score not specified)	**34.7%**							
LB‐MCI (9 studies)														
*Donaghy 2017* ^32^ *England*	DLB, **LB‐MCI**, AD, AD‐MCI, HC McKeith 2005	Inclusion: MMSE > 11 Exclusion: major concurrent psychiatric illness; severe physical illness; other significant neurological illness; current treatment with any investigational agent	36, 66.7%	75.3 (7.6)	MMSE 26.3 (2.3)	The Lewy Body Dementia Association Com‐prehensive Checklist	**47.2%**							
Donaghy 2018^56^ England	**LB‐MCI**, AD‐MCI Albert et al. 2011	Inclusion: age > 59 years Exclusion: dementia, MMSE < 20, CDR > 0.5, parkinsonism developing < 1 prior to cog impairment, clinical stroke, serious neurological or medical condition that would affect performance in study assessments	41, 34.1%	75.5 (7.6)	CDR‐GS 0.5 [0.5–0.5]	NPI (cut‐off score not specified)	**53.7%**							
*Liu 2020* ^57^ *China*	DLB, **LB‐MCI**, AD, AD‐MCI McKeith 2017	Exclusion: mixed dementia etiology (e.g., severe hippocampal atrophy in LB‐MCI), major concurrent psychiatric illness or a history of other significant neurological illness, including stroke or alcohol/substance abuse history, lack of caregiver	53, 56.6%	69 [65 – 77.5]	MMSE 25 [22–27] MoCA 18 [16–22]	NPI (cut‐off score not specified)	**39.6%**							
VanDeBeek 2020^58^ Netherlands	**LB‐MCI**, AD‐MCI McKeith 2017	None specified	73, 87.7%	67.9 (6.1)	MMSE 27 (2)	NPI (cut‐off score not specified)	**74%**							
*Galvin 2021* ^38^ *USA*	DLB, **LB‐MCI**, AD, AD‐MCI, HC McKeith 2020	None specified	22, 68.2%	75.3 (5.3)	CDR‐GS 0.6 (0.3) CDR‐SOB 1.9 (1.4)	NPI (cut‐off score not specified)	**45.5%**							
Donaghy 2022^59^ England	**LB‐MCI**, AD‐MCI McKeith 2020	Exclusion: dementia, MMSE < 20, CDR > 0.5, parkinsonism > 1 year prior to cog impairment clinical stroke or a serious neurological or medical condition, symptomatic heart failure (potential false‐positive cardiac MIBG results), current depression, history of bipolar disorder or schizophrenia	28, 96.4%	74.6 (5.7)	MMSE 26.3 (2.4) CDR‐GS 0.5 [0.5–0.5]	NPI (cut‐off score not specified)	**67.9%**							
*Gan 2022* ^40^ *China*	DLB, **LB‐MCI**, HC McKeith 2020	Exclusion: disagreement in diagnosis; other neurological disease; currently using orexin receptor antagonists; history of mental disorders and illicit drug abuse; acute or chronic liver and kidney dysfunction, malignant tumors, or other serious underlying disease	41, 12.5%	70.83 (5.96)	MMSE 20.07 (2.17) MoCA 18.12 (0.56) CDR‐GS 0.5 (0)	NPI (cut‐off score not specified)	**17.1%**	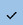						
*VanDeBeek 2021* ^39^ *Netherlands*	DLB, **LB‐MCI** McKeith 2020	Inclusion: MMSE > 19 or CDR, 1, available baseline high resolution MRI Exclusion: physical life threatening conditions, nursing home	27, 92.6%	67 (7)	MMSE 27 (2)	NPI (apathy subscale frequency × severity > 0)	**50%**							
Ting 2023^60^ Singapore	**LB‐MCI**, AD‐MCI McKeith 2005 (neuro‐pathological)	Exclusion: Neurofibrillary degeneration of Braak stages V and VI; PDD	111, 75.7%	76.9 (8.5)	MMSE 27.5 (2.2)	NPI (cut‐off score not specified)	**25%**							
PD‐MCI (5 studies)														
*Leroi 2012* ^49^ *UK*	PDD, **PD‐MCI**, PD MDS Task Force 2012	None specified	48, 70.8%	68.6 (8.4)	MMSE 27.64 (1.9)	NPI (apathy subscale frequency × severity > 0)	**47.9%**	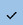						
Monastero 2013^61^ Italy	**PD‐MCI** (amnestic [a‐] and non‐amnestic [na‐] PD‐MCI) Peter‐son's criteria	Exclusion: severe systemic disorder; psychosis; history of significant head injury or substance abuse; dementia, according to DSM‐IV criteria	246, 59.8%	aMCI 69.9 (8.7); naMCI 71 (8.5)	MMSE aMCI 26.3 (1.9) naMCI 27 (2)	NPI (apathy subscale frequency × severity > 0)	**51.2%**	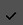						
Baschi 2020^62^ Italy	**PD‐MCI**, PD, MCI (not associated with PD) MDS Task Force 2007	Inclusion: mild‐moderate PD (e.g., Hoehn and Yahr Stage I to III) exclusion: presence of significant depression, PDD	31, 64.5%	66.7 (14.7)	MMSE 26.1 (2)	NPI (cut‐off score not specified)	**29%**	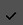						
Giguere‐Rancourt 2021^63^ Canada	**PD‐MCI** MDS Task Force 2012	Inclusion: MoCA scores 21 – 27, anti‐Parkinson medication stable at screening for at least 2 months, all other medications, including psychotropics, stable for at least 3 months. Exclusion: dementia, other neurological or psychiatric disorders	12, 83.3%	70.5 (5.35)	MoCA 23.9 (2.15)	NPI (cut‐off score not specified)	**25%**							
Lee 2023^64^ South Korea	**PD‐MCI** Neuro‐psycho‐logical testing	Inclusion: dopaminergic depletion in the posterior putamen on FP‐CIT PET	388, 45.6%	Those that didn't convert to PDD: 69.7 (7.7); those that did convert to PDD 71.5 (8.2)	Those that didn't convert to PDD: 26.7 (2.2); those that did convert to PDD 24.8 (3.6)	NPI (apathy subscale frequency × severity > 0)	**25.5%** Those that didn't covert to PDD: 28.3%; those that did convert to PDD: 36.6%	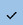						
LBD (2 studies)														
Kushwaha 2017^65^ India	**LBD**, AD, VaD, Mixed, FTD McKeith 2005, DSM IV	None specified	214, 57%	62 (range 60 – 77)	MMSE 14.7 (9.9)	NPI (cut‐off score not specified)	**0%**							
Borda 2023^66^ Norway	**LBD**, AD McKeith 2017, MDS Task Force 2007	Inclusion: MMSE score ≥ 20 or a CDR‐GS, 1	85, 55.3%	75.42 (6.9)	MMSE 23.65 (3.20)	NPI (cut‐off score not specified)	**51.2%**		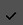					

*Note*: *Italicized articles* report more than one diagnostic group of interest and hence appear more than once in the table.

Abbreviations: AD, Alzheimer's disease; AES, Apathy Evaluation Scale; AES‐I, Apathy Evaluation Scale—Informant Questionnaire; BPSD, behavioral and psychological symptoms of dementia; CDR‐GS, Clinical Dementia Rating Scale Global Score; CDR‐SOB, Clinical Dementia Rating Scale Sum of Boxes; CNS, central nervous system; CT, computed tomography; DLB, dementia with Lewy bodies; DSM—III, IV, Diagnostic and Statistical Manual of Mental disorders version 4 and 5; FTD, frontotemporal dementia; FP‐CIT, N‐3‐[^18^F]‐fluoropropyl‐2β‐carbomethoxy‐3β‐(4‐iodophenyl) nortropane; HC, healthy control; IQR, interquartile range; LARS, Lille Apathy Scale; LBD, Lewy body dementia; MCI, mild cognitive impairment; MDS, Movement Disorders Society; MIBG, ^123^I‐metaiodobenzylguanidine; MMSE, Mini‐Mental State Examination; MoCA, Montreal Cognitive Assessment; MRI, magnetic resonance imaging; NPI, Neuropsychiatric Inventory; PD, Parkinson's disease; PDD, Parkinson's disease dementia; PET, positron emission tomography; SCOPA‐Cog, Scale for Outcomes in Parkinson's Disease—Cognition; SD, standard deviation; VaD, vascular dementia.

Clinical correlates of apathy:

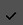
Apathy is associated with worse outcomes in clinical correlates.

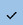
No significant association/correlation was found between apathy and clinical correlates.

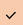
Apathy is associated with better outcomes in clinical correlates.

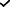
Clinical correlate assessed.

Blank, clinical correlate not assessed.

### 3.2 Study quality assessment

The quality of the articles included in this study was assessed to be generally good by two independent reviewers (Appendix B in supporting information). The main limitations found were that some studies did not specify whether they recruited convenience or consecutive samples, and some sample frames addressed a specific target population, which may not be representative of the general population (e.g., clinical trials).[Table alz70425-tbl-0001], [Table alz70425-tbl-0002], [Fig alz70425-fig-0003], [Fig alz70425-fig-0004]

### 3.3 Prevalence of apathy in LBD

**FIGURE 3 alz70425-fig-0003:**
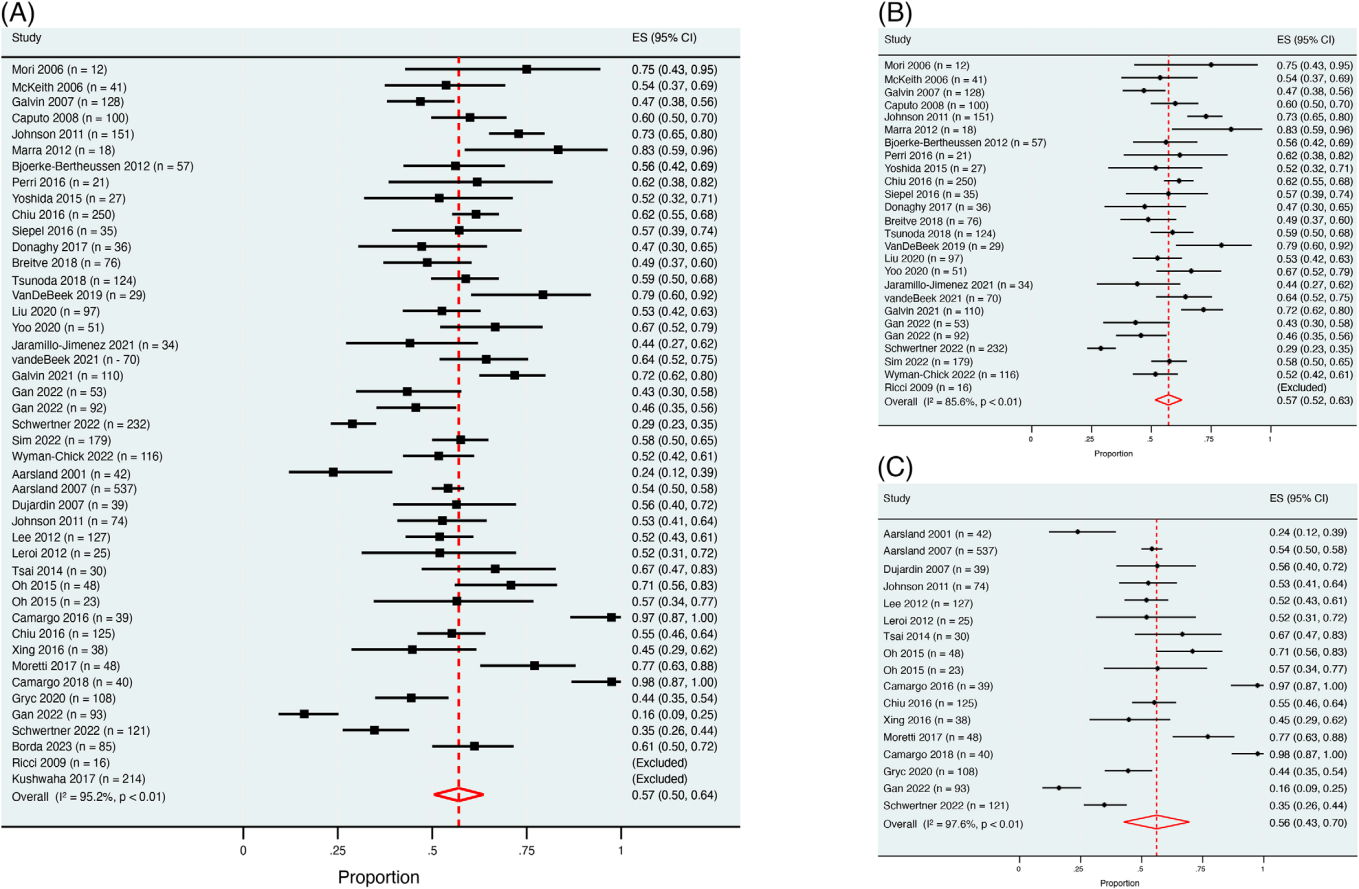
Average pooled prevalence of apathy in LBD (A), DLB (B), and PDD (C). CI, confidence interval; DLB, dementia with Lewy bodies; ES, effect size; LBD, Lewy body dementia; PDD, Parkinson's disease dementia

**FIGURE 4 alz70425-fig-0004:**
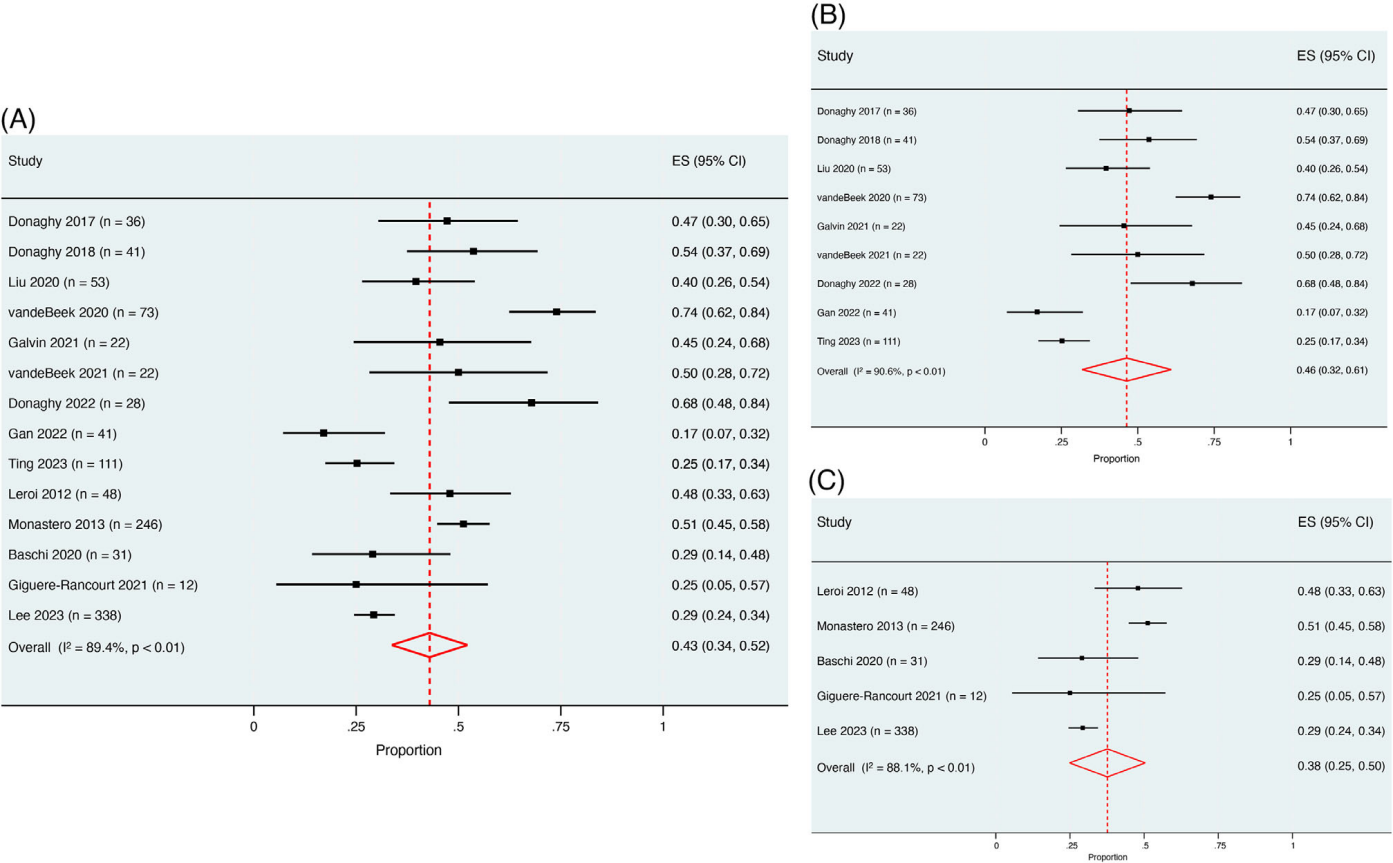
Average pooled prevalence of apathy in combined MCI (A), LB‐MCI (B), and PD‐MCI (C). CI, confidence interval; ES, effect size; LB‐MCI, Lewy body mild cognitive impairment; PD‐MCI, Parkinson's disease with mild cognitive impairment

The average pooled prevalence of apathy by diagnostic group is summarized in Table 2. The prevalence of apathy in LBD (DLB and PDD, *n* = 4011) was 57% (95% CI 50%–64%, *I* ^2^ = 95.2%; Figure 3A). The prevalence of apathy in DLB (*n* = 2155) and PDD (*n* = 1557) were 57% (95% CI 52%–63%, *I* ^2^ = 85.6%) and 56% (95% CI 43%–70%, *I* ^2^ = 97.6%), respectively (Figures 3B and C). The pooled prevalence of apathy in combined MCI groups (*n* = 1102) was 43% (95% CI 34%– 52%, *I* ^2^ = 89.4%; Figure 4A). The prevalence in LB‐MCI (*n* = 427) and PD‐MCI (*n* = 675) was 46% (95% CI 32%– 61%, *I* ^2^ = 90.6%) and 38% (95% CI 25%– 50%, *I* ^2^ = 88.1%), respectively (Figures 4B and C). Reported prevalences by individual studies varied considerably. Of note, the lowest prevalence reported in DLB (28.9%) was by Schwertner's group,^42^ who focused on residential care residents. Prevalence > 80% was reported by Marra et al.^27^ (83.3%) and Ricci et al.^26^ (100%). Both these DLB studies were small community‐dwelling cohorts (*n* < 20). Similar variability was observed between PDD studies. Notably, Camargo's group used the Brazilian version of the AES cut‐off of > 14 to diagnose apathy in both their studies and found a prevalence rate of > 97%.^9^, ^52^

**TABLE 2 alz70425-tbl-0002:** Pooled prevalence of apathy by diagnostic group.

Diagnosis group (total participants)	Pooled prevalence (95% CI), *I* ^2^
LBD (*n*, 4011)	57% (50%–64%), 95.2%
DLB (*n*, 2155)	57% (52%–63%), 85.6%
PDD (*n*, 1557)	56% (43%–70%), 97.6%
Combined MCI (*n*, 1102)	43% (34%–52%), 89.4%
LB‐MCI (*n*, 427)	46% (32%–61%), 90.6%
PD‐MCI (*n*, 675)	38% (25%–50%), 88.1%

Abbreviations: CI, confidence interval; DLB, dementia with Lewy bodies; LBD, Lewy body dementia; MCI, mild cognitive impairment; PD, Parkinson's disease; PDD, Parkinson's disease dementia.

Seven studies used cohorts (DLB, PDD, and LB‐MCI) that overlapped with those of other studies included in the review. To account for this, these studies were removed, and a sensitivity analysis was performed on the remaining group. The pooled apathy prevalence estimates in the remaining studies were similar to the whole group: 55% (95% CI 49%–62%, *I* ^2^ = 94.4%) in LBD (*n* = 3709), 57% (95% CI 51%–63%, *I* ^2^ = 87.1%) in DLB (*n* = 2000), 53% (95% CI 40%–66%, *I* ^2^ = 97%) in PDD (*n* = 1495), 42% (95% CI 31%–52%, *I* ^2^ = 89.7%) in combined MCI (*n* = 902), and 46% (95% CI 28%–64%, *I* ^2^ = 92.9%) in LB‐MCI (*n* = 369; Figures S1–S5 in supporting information).

Twenty‐two studies reported sample sizes > 50. They included 3370 LBD participants, 1886 DLB participants, 1185 PDD participants, and 821 combined MCI participants (237 LB‐MCI and 584 PD‐MCI). Post hoc sensitivity analysis of these studies demonstrated similar prevalence estimates: 52% (95% CI 47%–58%, *I* ^2^ = 91.1%) in LBD, 55% (95% CI 49%–62%, *I* ^2^ = 89.3%) in DLB, 44% (95% CI 33%–55%, *I* ^2^ = 93.3%) in PDD, and 44% (95% CI 28%–60%, *I* ^2^ = 95.4%) in the combined MCI group (Figures S6–S9 in supporting information).

Last, post hoc sensitivity analysis was performed in the 43 articles that used NPI as their apathy assessment tool (Figures S10–S14 in supporting information). The prevalence of apathy were similar: 55% (95% CI 50%–60%, *I* ^2^ = 89.2%) in LBD (*n* = 3678), 58% (95% CI 52%–64%, *I* ^2^ = 87%) in DLB (*n* = 1940), 50% (95% CI 41%–59%, *I* ^2^ = 91.1%) in PDD (*n* = 1439), 43% (95% CI 33%–52%, *I* ^2^ = 90.1%) in the combined MCI group (*n* = 1066), and 46% (95% CI 30%–62%, 91.8%) in LB‐MCI (*n* = 391).

### 3.4 Assessment of clinical correlates of apathy in LBD in the included articles

Eighteen of the 49 articles included in our systematic review reported clinical correlates of apathy, including cognition (11 articles), function (3 articles), motor impairment (5 articles), quality of life (2 articles), residental care admission (1 article), mortality (1 article), and caregiver burden (2 articles). Given the variation in study design, including outcomes of interest, meta‐analysis of clinical correlates was not possible.

#### 3.4.1 Assessment variability

Cognition was measured using cognitive tests (MMSE, Scale for Outcomes in Parkinson's Disease—Cognition [SCOPA‐COG]), structured interviews that assess dementia severity (CDR), or defined by the presence of dementia (MCI vs. dementia). Function was measured in DLB cohorts only, using three different tools (Katz Activities of Daily Living, Instrumental Activities of Daily Living, and Functional Activities Questionnaire). Motor impairment, but not function, was investigated in PDD cohorts using two scales (UPDRS III and H & Y). Quality of life was measured in two articles using three different scales: EuroQol‐5D, Visual Analogue Scale, and Quality of Life in Alzheimer's Disease scale. Caregiver burden was similarly only reported by two studies using three different scales: Zarit's Burden Interview, Burden Interview, and Caregiver Burden Inventory.

#### 3.4.2 Analysis variability

The statistical methods used to describe the relationships between clinical correlates of apathy were similarly variable. Some articles dichotomized participants into groups based on the clinical correlate of interest (presence vs. absence of dementia or by cognitive testing or motor impairment scores) and compared apathy prevalence and severity between the two groups. Others interrogated the correlation between apathy and clinical correlates or used regression analyses to determine the contribution of apathy to clinical correlates.

Two articles reported longitudinal clinical correlates data. Borda et al.^66^ enrolled 85 LBD participants at baseline, with 20 completing a 5‐year follow‐up. There was no attrition data available for Breitve et al.’s cohort^6^ (baseline *n* = 76).

## 4 DISCUSSION

Our systematic review and meta‐analysis examined the prevalence of apathy in LBD. We demonstrated an average pooled apathy prevalence of 57% in LBD (57% in DLB, 56% in PDD), 46% in LB‐MCI, and 38% in PD‐MCI. These findings underscore the significance of apathy as a common syndrome across the disease continuum.

We found a wide range of apathy prevalences reported in individual studies (from < 30%^41^, ^42^ to > 80%^9^, ^26^, ^27^, ^52^). Small sample sizes (median sample size 51) may account for some of this variation. However, results of our sensitivity analysis of studies reporting cohorts of > 50 were similar to the pooled prevalence of all included studies. Another reason may relate to the assessment tools used. The most commonly used tool was the NPI, which was originally developed in 1997 for use in dementia, mostly using AD cohorts.^67^ The NPI includes a wide range of neuropsychiatric symptoms, asking the caregiver initially to provide a “yes” or “no” answer to each potential symptom. If the caregiver answers “no,” no further examples of how the symptom manifests are provided. This may lead to under‐reporting from those who may not recognize apathy without clinical examples. In contrast, the 18‐question AES and 33‐question LARS, validated in AD and stroke^68^ and PD cohorts, respectively,^69^ are solely focused on apathy, and can further characterize apathy by its various dimensions (behavioral, cognitive, and emotional apathy), which is not only a requirement for apathy diagnosis,^1^ but may also have research^70^ and clinical utility. This approach is therefore potentially more accurate and sensitive but may be prone to misinterpretation by caregivers, as was suggested by Camargo's group, who reported apathy prevalence of > 97% in two PDD studies^9^, ^52^ using the Brazilian AES. Given so few of the included articles used tools other than the NPI, it is unsurprising that our sensitivity analysis of the 43 articles using NPI demonstrated similar results to the pooled prevalence of all included studies. In keeping with the findings on sensitivity analyses, the high *I* ^2^ values of >r 85% in all diagnostic groups means, by definition, that at least 85% of the variability of prevalence estimates seen in the included studies would remain if sampling error, such as selection based on dementia severity, were eliminated.^21^ Other sources of variability may also include differences in study methodology and diagnostic variability.

We found that some of the lowest apathy rates (28.9% in DLB and 34.7% in PDD) were reported by one study using a registry of residential care facilities.^42^ This was the only study in our review that included a specific residential care cohort. The most common neuropsychiatric symptoms in all dementia types in this study were instead aberrant motor behavior, agitation/aggression, and irritability. Hence, one reason for the low apathy rate may be selection bias, as individuals who have challenging “active” behaviors may disproportionately enter residential care. However, this would not be in keeping with previous findings that apathy is a source of significant burden for family caregivers,^71^, ^72^ which may also lead to residential care entry. Another consideration in residential care settings may be underreporting or under‐recognition of apathy as formal caregivers regard apathy as one of the least distressing symptoms to manage day to day^73^ and have reported challenges in identifying and appreciating the significance of apathy.^74^ However, low rates of apathy are not seen universally in residential aged care settings. A large Dutch study of apathy in residential care that did not distinguish between dementia types^75^ demonstrated apathy prevalence of 52%, which is more consistent with our overall meta‐analysis finding. Future studies should consider the influence of residential setting, including staff awareness, on apathy in LBD and how it may impact residents’ outcomes.

We found that almost half (46%) of individuals with prodromal DLB (LB‐MCI) had apathy. Previous studies have reported higher rates of apathy in prodromal DLB (between 50% and 70%) compared to prodromal AD (< 20%), and demonstrated high sensitivity and specificity of apathy for differentiating between future DLB versus AD diagnosis.^32^, ^56^, ^59^ Approximately two thirds of individuals who are later confirmed to have DLB at *post mortem* are diagnosed with AD during life,^76^ making AD the main clinical differential for DLB. Taken together, these findings suggest that apathy may assist in earlier and more accurate diagnosis of DLB, differentiating it from AD. Further work in larger prospective cohorts is required to confirm these results.

Similarly, we found that apathy prevalence was higher in prodromal DLB compared to PD‐MCI (46% vs. 38%, respectively). This may suggest that the earlier involvement of limbic structures in addition to the substantia nigra and striatum, seen in prodromal DLB compared to PD,^77^ contributes to the development of apathy in LBD.

Finally, apathy in LBD is associated with several adverse clinical outcomes for individuals and their caregivers. The variable methods of measuring clinical correlates, such as cognition, function, quality of life, and caregiver outcomes in the studies we identified, highlight the need for standardized outcome measures in DLB research. The articles we reviewed also lacked longitudinal data on clinical correlates, which would be needed to better understand the prognostic significance of apathy and the long‐term relationship between apathy and clinical outcomes.

Our study has some limitations. There was heterogeneity in the studies we included, highlighting the imprecision and variability of LBD research methods. This made comparing and aggregating prevalence estimates challenging, resulting in large CIs and high heterogeneity (*I* ^2^) values. However, it is worth noting that high *I* ^2^ values are commonly seen in meta‐analyses of proportion, especially those which include a large number of studies reporting highly variable proportions, and therefore should not be relied upon as the sole indicator of heterogeneity.^78^ One source of heterogeneity in our study includes the small median sample size of our studies, although reassuringly, the sensitivity analysis of studies with a sample size of ≥ 50 produced similar results. Another source of heterogeneity was variability in dementia stage. We were unable to incorporate this into our analysis as dementia stage was not reported uniformly in the included studies (e.g., studies variably reported MMSE, MoCA, CDR, or other measures). Many of the included studies used convenience rather than consecutive sampling. This may introduce a degree of selection bias, especially as individuals who consent to participating in research are potentially more motivated than the general LBD population, leading to an underrepresentation of true apathy prevalence. Convenience sampling can also limit research participation from culturally and linguistically diverse and lower socioeconomic groups. These factors may affect apathy in LBD in various ways, which were not explored. Publication bias is also an important consideration in meta‐analysis. Importantly, the majority of the included studies were not primarily assessing apathy prevalence; hence, very high or very low prevalence should not have influenced the publication of these studies.

## 5 CONCLUSION

Apathy is an important syndrome that affects at least 1 in 2 people with LBD. It is common in both prodromal (especially prodromal DLB) and dementia stages of the disease continuum. The high prevalence of apathy in prodromal DLB supports the notion that apathy is a specific feature of emerging DLB, with potential implications for future diagnostic criteria. These interesting results should, however, be interpreted in the context of observed heterogeneity in study methodology and potential diagnostic variability in the studies included. The NPI is the most common tool used to detect apathy in clinical studies, although it does not assess the various dimensions of apathy and may not be as sensitive as other available tools. Although clinical correlates of apathy are often measured, the methods of measurement are variable, meaning results from different studies are not easily compared. There is a lack of longitudinal data regarding clinical correlates of apathy, which is needed to better understand associations with clinical outcomes. Future research should prospectively investigate apathy as part of an early detection tool for DLB, consider using dedicated apathy assessment tools which can delineate the various dimensions of apathy, as well as focus on characterizing apathy and its clinical correlates in longitudinal LBD cohorts.

## CONFLICT OF INTEREST STATEMENT

J.J.Y., K.S.C., P.M.L., L.C., S.M.L., N.Y., and R.W. declare no conflicts of interest. Author disclosures are available in the supporting information.

## CONSENT STATEMENT

Consent was unnecessary for this study.

## Supporting information



Supporting Information

Supporting Information

Supporting Information

## Data Availability

Data collection template, data extracted from included studies, and data used for all analyses can be made available upon reasonable request, including a data sharing agreement.

## References

[1] Miller DS , Robert P , Ereshefsky L , et al. Diagnostic criteria for apathy in neurocognitive disorders. Alzheimers Dement. 2021;17(12):1892‐1904. doi:10.1002/alz.12358 33949763 PMC8835377

[2] Burchill E , Watson CJ , Fanshawe JB , et al. The impact of psychiatric comorbidity on Parkinson's disease outcomes: a systematic review and meta‐analysis. Lancet Reg Health Eur. 2024;39:100870. doi:10.1016/j.lanepe.2024.100870 38361749 PMC10867667

[3] McKeith IG , Ferman TJ , Thomas AJ , et al. Research criteria for the diagnosis of prodromal dementia with Lewy bodies. Neurology. 2020;94(17):743‐755. doi:10.1212/wnl.0000000000009323 32241955 PMC7274845

[4] McKeith IG , Boeve BF , Dickson DW , et al. Diagnosis and management of dementia with Lewy bodies: fourth consensus report of the DLB Consortium. Neurology. 2017;89(1):88‐100. doi:10.1212/wnl.0000000000004058 28592453 PMC5496518

[5] Wyman‐Chick KA , O'Keefe LR , Weintraub D , et al. Prodromal dementia with Lewy bodies: evolution of symptoms and predictors of dementia onset. J Geriatr Psychiatry Neurol. 2022;35(4):527‐534. doi:10.1177/08919887211023586 34114509 PMC9150711

[6] Breitve MH , Chwiszczuk LJ , Rongve A , Bronnick K , Hynninen MJ , Aarsland D . Apathy is associated with faster global cognitive decline and early nursing home admission in dementia with Lewy bodies. Alzheimers Res Ther. 2018;10(1):83. doi:10.1186/s13195-018-0416-5 30121084 PMC6098842

[7] Caputo M , Mariani E , Santucci A , et al. Neuropsychiatric symptoms in 921 elderly subjects with dementia: a comparison between vascular and neurodegenerative types. Acta Psychiatr Scand. 2008;117(6):455‐464. doi:10.1111/j.1600-0447.2008.01175.x 18363771

[8] Bjoerke‐Bertheussen J , Aarsland D , Rongve A , Ehrt U , Ballard C . Neuropsychiatric symptoms in mild dementia with Lewy bodies and Alzheimer's disease. Dement Geriatr Cogn Disord. 2012;34(1):1‐6. doi:10.1159/000339590 22854420

[9] Camargo CHF , Serpa RA , Jobbins VA , Berbetz FA , Sabatini JS . Differentiating between apathy and depression in patients with Parkinson disease dementia. Am J Alzheimers Dis Other Demen. 2018;33(1):30‐34. doi:10.1177/1533317517728333 28871794 PMC10852426

[10] Johnson DK , Chapin BA , Anderson R , Watts AS , Burns JM . Neuropsychiatric profiles in dementia. Alzheimer Dis Assoc Disord. 2011;25(4):326‐332. doi:10.1097/WAD.0b013e31820d89b6 22086220 PMC3218373

[11] Nagahama Y , Okina T , Suzuki N , Matsuda M , Fukao K , Murai T . Classification of psychotic symptoms in dementia with Lewy bodies. Am J Geriatr Psychiatry. 2007;15(11):961‐967. doi:10.1097/JGP.0b013e3180cc1fdf 17974867

[12] Aarslan D , Litvan I , Larsen JP . Neuropsychiatric symptoms of patients with progressive supranuclear palsy and Parkinson's disease. J Neuropsychiatry Clin Neurosci. 2001;13(1):42‐49. doi:10.1176/jnp.13.1.42 11207328

[13] Abrantes AM , Friedman JH , Brown RA , et al. Physical activity and neuropsychiatric symptoms of Parkinson disease. J Geriatr Psychiatry Neurol. 2012;25(3):138‐145. doi:10.1177/0891988712455237 22914597

[14] Cubo E , Benito‐Leon J , Coronell C , Armesto D , Grp AS . Clinical correlates of apathy in patients recently diagnosed with Parkinson's disease: the ANIMO study. Neuroepidemiology. 2012;38(1):48‐55. doi:10.1159/000334314 22236943 PMC3325546

[15] Bronnick K , Aarsland D , Larsen JP . Neuropsychiatric disturbances in Parkinson's disease clusters in five groups with different prevalence of dementia. Acta Psychiatr Scand. 2005;112(3):201‐207. doi:10.1111/j.1600-0447.2005.00562.x 16095475

[16] Leung DKY , Wong GHY , Chan WC , Spector A . Prevalence of depression, anxiety, and apathy symptoms across dementia stages: a systematic review and meta‐analysis. Int J Geriatr Psychiatry. 2021;36(9):1330‐1344. doi:10.1002/gps.5556 33905138

[17] den Brok MG , van Dalen JW , van Gool WA , et al. Apathy in Parkinson's disease: a systematic review and meta‐analysis. Mov Disord. 2015;30(6):759‐769. doi:10.1002/mds.26208 25787145

[18] Emre M , Aarsland D , Brown R , et al. Clinical diagnostic criteria for dementia associated with Parkinson's disease. Mov Disord. 2007;22(12):1689‐1707. doi:10.1002/mds.21507 17542011

[19] Litvan I , Goldman JG , Tröster AI , et al. Diagnostic criteria for mild cognitive impairment in Parkinson's disease: movement Disorder Society Task Force guidelines. Mov Disord. 2012;27(3):349‐356. doi:10.1002/mds.24893 22275317 PMC3641655

[20] Munn Z , Moola S , Lisy K , Riitano D , Tufanaru C . Chapter 5: systematic reviews of prevalence and incidence. In: Aromataris E , Munn Z , eds. JBI Manual for Evidence Synthesis. JBI; 2020.

[21] Borenstein M , Higgins JP , Hedges LV , Rothstein HR . Basics of meta‐analysis: I^2^ is not an absolute measure of heterogeneity. Res Synth Methods. 2017;8(1):5‐18. doi:10.1002/jrsm.1230 28058794

[22] Higgins J , Thomas J , Chandler J , Cumpston M, Li T, Page MJ, Welch VA (editors). Cochrane Handbook for Systematic Reviews of Interventions. Version 6.5. Cochrane; 2024. www.training.cochrane.org/handbook 10.1002/14651858.ED000142 PMC10284251 31643080

[23] Mori E , Iseki E , Kosaka K , Mori S . Efficacy and safety of donepezil in patients with dementia with Lewy bodies: Preliminary findings from an open‐label study. Psychiatry Clin Neurosci. 2006;60(2):190‐195. doi:10.1111/j.1440-1819.2006.01485.x 16594943

[24] McKeith IG , Rowan E , Naidu A , et al. More severe functional impairment in dementia with Lewy bodies than Alzheimer disease is related to extrapyramidal motor dysfunction. Am J Geriatr Psychiatry. 2006;14(7):582‐588. doi:10.1097/01.JGP.0000216177.08010.f4 16816011

[25] Galvin JE , Malcom H , Johnson D , Morris JC . Personality traits distinguishing dementia with Lewy bodies from Alzheimer disease. Neurology. 2007;68(22):1895‐1901. doi:10.1212/01.wnl.0000263131.80945.ad 17536045

[26] Ricci M , Guidoni SV , Sepe‐Monti M , et al. Clinical findings, functional abilities and caregiver distress in the early stage of dementia with Lewy bodies (DLB) and Alzheimer's disease (AD). Arch Gerontol Geriatr. 2009;49(2):e101‐e104. doi:10.1016/j.archger.2008.10.001 19084284

[27] Marra C , Quaranta D , Profice P , et al. Central cholinergic dysfunction measured “in vivo” correlates with different behavioral disorders in Alzheimer's disease and dementia with Lewy body. Brain Stimul. 2012;5(4):533‐538. doi:10.1016/j.brs.2011.08.009 22019082

[28] Yoshida T , Mori T , Yamazaki K , et al. Relationship between regional cerebral blood flow and neuropsychiatric symptoms in dementia with Lewy bodies. Int J Geriatr Psychiatry. 2015;30(10):1068‐1075. doi:10.1002/gps.4263 25694273

[29] Chiu P‐Y , Tsai C‐T , Chen P‐K , Chen W‐J , Lai T‐J . Neuropsychiatric symptoms in Parkinson's disease dementia are more similar to Alzheimer's disease than dementia with lewy bodies: A case‐control study. PLoS One. 2016;11(4):e0153989. doi:10.1371/journal.pone.0153989 27101140 PMC4839640

[30] Perri R , Monaco M , Fadda L , Caltagirone C , Carlesimo GA . Neuropsychological Correlates of Behavioral Symptoms in Alzheimer's Disease, Frontal Variant of Frontotemporal, Subcortical Vascular, and Lewy Body Dementias: A Comparative Study. Focus (Am Psychiatr Publ). 2016;14(4):516‐522. doi:10.1176/appi.focus.140407 31997965 PMC6519600

[31] Siepel FJ , Dalen I , Gruner R , et al. Loss of Dopamine Transporter Binding and Clinical Symptoms in Dementia With Lewy Bodies. Mov Disord. 2016;31(1):118‐125. doi:10.1002/mds.26327 26207978

[32] Donaghy PC , Barnett N , Olsen K , et al. Symptoms associated with Lewy body disease in mild cognitive impairment. Int J Geriatr Psychiatry. 2017;32(11):1163‐1171. doi:10.1002/gps.4742 28556415

[33] Tsunoda N , Hashimoto M , Ishikawa T , et al. Clinical features of auditory hallucinations in patients with dementia with lewy bodies: A soundtrack of visual hallucinations. J Clin Psychiatry. 2018;79(3):17m11623. doi:10.4088/JCP.17m11623 29742332

[34] Van De Beek M , Van Steenoven I , Lemstra AW , et al. Trajectories and Determinants of Quality of Life in Dementia with Lewy Bodies and Alzheimer's Disease. J Alzheimers Dis. 2019;70(2):387‐395. doi:10.3233/JAD-190041 PMC6839497 31177218

[35] Liu B‐L , Li C‐I , Lane H‐Y , et al. Activation of N‐methyl‐D‐aspartate receptor glycine site temporally ameliorates neuropsychiatric symptoms of Parkinson's disease with dementia. Psychiatry Clin Neurosci. 2014;68(9):692‐700. doi:10.1111/pcn.12175 24612097

[36] Yoo HS , Chung SJ , Lee YH , et al. Clinical and striatal dopamine transporter predictors of beta‐Amyloid in dementia with Lewy bodies. Neurology. 2020;94(13):E1344‐E1352. doi:10.1212/WNL.0000000000009168 32086384

[37] Jaramillo‐Jimenez A , Giil LM , Tovar‐Rios DA , et al. Association Between Amygdala Volume and Trajectories of Neuropsychiatric Symptoms in Alzheimer's Disease and Dementia With Lewy Bodies. Front Neurol. 2021;12:679984. doi:10.3389/fneur.2021.679984 34305791 PMC8292611

[38] Galvin JE , Chrisphonte S , Cohen I , et al. Characterization of dementia with Lewy bodies (DLB) and mild cognitive impairment using the Lewy body dementia module (LBD‐MOD). Alzheimers Dis. 2021;17(10):1675‐1686. doi:10.1002/alz.12334 PMC8484363 33793069

[39] van de Beek M , van Steenoven I , van der Zande JJ , et al. Characterization of symptoms and determinants of disease burden in dementia with Lewy bodies: DEvELOP design and baseline results. Alzheimers Res Ther. 2021;13(1):53. doi:10.1186/s13195-021-00792-w 33637117 PMC7908769

[40] Gan JH , Liu S , Chen ZC , et al. Elevated Plasma Orexin‐A Levels in Prodromal Dementia with Lewy Bodies. J Alzheimers Dis. 2022;88(3):1037‐1048. doi:10.3233/jad-220082 35723094

[41] Gan J , Chen Z , Liu S , et al. The presence and co‐incidence of geriatric syndromes in older patients with mild‐moderate Lewy body dementia. BMC Neurol. 2022;22(1):355. doi:10.1186/s12883-022-02897-7 36123648 PMC9484208

[42] Schwertner E , Pereira JB , Xu H , et al. Behavioral and psychological symptoms of dementia in different dementia disorders: a large‐scale study of 10,000 individuals. J Alzheimers Dis. 2022;87(3):1307‐1318. doi:10.3233/jad-215198 35491774 PMC9198804

[43] Sim J , Li HH , Hameed S , Ting SKS . Clinical Manifestations of Early‐Onset Dementia With Lewy Bodies Compared With Late‐Onset Dementia With Lewy Bodies and Early‐Onset Alzheimer Disease. JAMA Neurol. 2022;79(7):702‐709. doi:10.1001/jamaneurol.2022.1133 35604656 PMC9127709

[44] Wyman‐Chick KA , O'Keefe LR , Rosenbloom M , et al. Prodromal Dementia With Lewy Bodies: Evolution of Symptoms and Predictors of Dementia Onset. J Geriatr Psychiatry Neurol. 2021;doi:10.1177/08919887211023586 PMC9150711 34114509

[45] Aarsland D , Cummings JL , Larsen JP . Neuropsychiatric differences between Parkinson's disease with dementia and Alzheimer's disease. Int J Geriatr Psychiatry. 2001;16(2):184‐191. doi:10.1002/1099-1166(200102)16:2<184::AID-GPS304>3.0.CO;2-K 11241724

[46] Aarsland D , Bronnick K , Ehrt U , et al. Neuropsychiatric symptoms in patients with Parkinson's disease and dementia: Frequency, profile and associated care giver stress. J Neurol Neurosurg Psychiatry. 2007;78(1):36‐42. doi:10.1136/jnnp.2005.083113 16820421 PMC2117797

[47] Dujardin K , Sockeel P , Devos D , et al. Characteristics of apathy in Parkinson's disease. Mov Disord. 2007;22(6):778‐784. doi:10.1002/mds.21316 17290451

[48] Lee W‐J , Tsai C‐F , Gauthier S , Wang S‐J , Fuh J‐L . The association between cognitive impairment and neuropsychiatric symptoms in patients with Parkinson's disease dementia. Int Psychogeriatr. 2012;24(12):1980‐1987. doi:10.1017/S1041610212001317 22835209

[49] Leroi I , Pantula H , McDonald K , Harbishettar V . Neuropsychiatric symptoms in parkinsons disease with mild cognitive impairment and dementia. Parkinsons Dis. 2012:308097. doi:10.1155/2012/308097 22970412 PMC3437302

[50] Oh YS , Lee JE , Lee PH , Kim JS . Neuropsychiatric symptoms in Parkinson's disease dementia are associated with increased caregiver burden. J Mov Disord. 2015;8(1):26‐32. doi:10.14802/jmd.14019 25614783 PMC4298716

[51] Oh YS , Kim JS , Lee PH . Effect of Rivastigmine on Behavioral and Psychiatric Symptoms of Parkinson's Disease Dementia. J Mov Disord. 2015;8(2):98‐102. doi:10.14802/jmd.15041 26090082 PMC4460546

[52] Camargo CHF , Serpa RA , Matnei T , Sabatini JS , Teive HAG . The perception of apathy by caregivers of patients with dementia in Parkinson's disease. Dement Neuropsychol. 2016;10(4):339‐343. doi:10.1590/s1980-5764-2016dn1004014 29213479 PMC5619275

[53] Xing Y , Tang Y , Zhao L , et al. Associations between plasma ceramides and cognitive and neuropsychiatric manifestations in Parkinson's disease dementia. J Neurol Sci. 2016;370:82‐87. doi:10.1016/j.jns.2016.09.028 27772793

[54] Moretti R , Caruso P , Dal Ben M . Rivastigmine as a symptomatic treatment for apathy in Parkinson's dementia complex: New aspects for this riddle. Parkinsons Dis. 2017;2017:6219851. doi:10.1155/2017/6219851 28409049 PMC5376458

[55] Gryc W , Roberts KA , Zabetian CP , et al. Hallucinations and Development of Dementia in Parkinson's Disease. J Parkinsons Dis. 2020;10(4):1643‐1648. doi:10.3233/JPD-202116 32741842 PMC7609584

[56] Donaghy PC , Taylor J‐P , Barnett N , et al. Neuropsychiatric symptoms and cognitive profile in mild cognitive impairment with Lewy bodies. Psychol Med. 2018;48(14):2384‐2390. doi:10.1017/S0033291717003956 29362011

[57] Liu C , Wang X , Ji Y , Liu S . Neuropsychiatric profiles in mild cognitive impairment with Lewy bodies. Aging Ment Health. 2020:1‐7. doi:10.1080/13607863.2020.1817311 32896168

[58] van de Beek M , van Steenoven I , van der Zande JJ , et al. Prodromal Dementia With Lewy Bodies: Clinical Characterization and Predictors of Progression. Mov Disord. 2020;35(5):859‐867. doi:10.1002/mds.27997 32048343 PMC7317511

[59] Donaghy PC , Ciafone J , Durcan R , et al. Mild cognitive impairment with Lewy bodies: neuropsychiatric supportive symptoms and cognitive profile. Psychol Med. 2022;52(6):1147‐1155. doi:10.1017/s0033291720002901 32840196

[60] Ting SKS , Saffari SE , Hameed S , Chiew HJ , Ng KP , Ng AS . Clinical characteristics of pathological confirmed prodromal dementia with Lewy bodies. J Neurol Sci. 2023;453(6):120815. doi:10.1016/j.jns.2023.120815 37757638 PMC10591830

[61] Monastero R , Di Fiore P , Ventimiglia GD , Camarda R , Camarda C . The neuropsychiatric profile of Parkinson's disease subjects with and without mild cognitive impairment. J Neural Transm. 2013;120(4):607‐611. doi:10.1007/s00702-013-0988-y 23400362

[62] Baschi R , Caccamo M , Di Giorgi L , et al. Changes in Motor, Cognitive, and Behavioral Symptoms in Parkinson's Disease and Mild Cognitive Impairment During the COVID‐19 Lockdown. Front Psychiatry. 2020;11:590134. doi:10.3389/fpsyt.2020.590134 33381057 PMC7768013

[63] Giguere‐Rancourt A , Plourde M , Racine E , et al. Altered Theory of Mind in Parkinson's Disease and Impact on Caregivers: A Pilot Study. Can J Neurol Sci. 2021. 49(3), 437–440. doi:10.1017/cjn.2021.110 33988099

[64] Lee YG , Park M , Jeong SH , et al. Association of neuropsychiatric symptom profiles with cognitive decline in patients with Parkinson disease and mild cognitive impairment. Empirical Study; Quantitative Study. Neurology. 2023;101(12):e1186‐e1195. doi:10.1212/WNL.0000000000207623 37524535 PMC10516268

[65] Kushwaha S , Anthony A , Bala K , et al. Clinical Spectrum, Risk Factors, and Behavioral Abnormalities among Dementia Subtypes in a North Indian Population: A Hospital‐Based Study. Dement Geriatr Cogn Dis Extra. 2017;7(2):257‐273. doi:10.1159/000478978 29033972 PMC5624266

[66] Borda MG , Bronnick KK , Garcia‐Cifuentes E , et al. Specific neuropsychiatric symptoms are associated with functional decline trajectories in Alzheimer's disease and Lewy body dementia: a five‐year follow‐up study. Front Med. 2023;10:1267060. doi:10.3389/fmed.2023.1267060 PMC10616879 37915329

[67] Cummings JL . The Neuropsychiatric Inventory: assessing psychopathology in dementia patients. Neurology. 1997;48(5 Suppl 6):S10‐S16. doi:10.1212/wnl.48.5_suppl_6.10s 9153155

[68] Marin RS , Biedrzycki RC , Firinciogullari S . Reliability and validity of the Apathy Evaluation Scale. Psychiatry Res. 1991;38(2):143‐162. doi:10.1016/0165-1781(91)90040-v 1754629

[69] Sockeel P , Dujardin K , Devos D , Deneve C , Destee A , Defebvre L . The Lille apathy rating scale (LARS), a new instrument for detecting and quantifying apathy: validation in Parkinson's disease. J Neurol Neurosurg Psychiatry. 2006;77(5):579‐584. doi:10.1136/jnnp.2005.075929 16614016 PMC2117430

[70] Cipriani G , Lucetti C , Danti S , Nuti A . Apathy and dementia. Nosology, assessment and management. J Nerv Ment Dis. 2014;202(10):718‐724. doi:10.1097/NMD.0000000000000190 25265266

[71] Chang CYM , Baber W , Dening T , Yates J . “He just doesn't want to get out of the chair and do it”: the impact of apathy in people with dementia on their carers. Int J Environ Res Public Health. 2021;18(12):6317. doi:10.3390/ijerph18126317 34207955 PMC8296153

[72] García‐Martín V , de Hoyos‐Alonso MC , Delgado‐Puebla R , Ariza‐Cardiel G , del Cura‐González I . Burden in caregivers of primary care patients with dementia: influence of neuropsychiatric symptoms according to disease stage (NeDEM project). BMC Geriatrics. 2023;23(1):525. doi:10.1186/s12877-023-04234-0 37644410 PMC10463529

[73] Zwijsen SA , Kabboord A , Eefsting JA , et al. Nurses in distress? An explorative study into the relation between distress and individual neuropsychiatric symptoms of people with dementia in nursing homes. Int J Geriatr Psychiatry. 2014;29(4):384‐391. doi:10.1002/gps.4014 23963653

[74] Nijsten JMH , Smalbrugge M , Plouvier AOA , Koopmans RTCM , Leontjevas R , Gerritsen DL . Identifying and managing apathy in people with dementia living in nursing homes: a qualitative study. BMC Geriatr. 2023;23(1):727. doi:10.1186/s12877-023-04422-y 37946109 PMC10636808

[75] Nijsten JMH , Leontjevas R , Pat‐El R , Smalbrugge M , Koopmans R , Gerritsen DL . Apathy: risk factor for mortality in nursing home patients. J Am Geriatr Soc. 2017;65(10):2182‐2189. doi:10.1111/jgs.15007 28791690

[76] Nelson PT , Jicha GA , Kryscio RJ , et al. Low sensitivity in clinical diagnoses of dementia with Lewy bodies. J Neurol. 2010;257(3):359‐366. doi:10.1007/s00415-009-5324-y 19795154 PMC2839040

[77] Outeiro TF , Koss DJ , Erskine D , et al. Dementia with Lewy bodies: an update and outlook. Mol Neurodegener. 2019;14(1):5. doi:10.1186/s13024-019-0306-8 30665447 PMC6341685

[78] Migliavaca CB , Stein C , Colpani V , et al. Meta‐analysis of prevalence: I^2^ statistic and how to deal with heterogeneity. Res Synth Methods. 2022;13(3):363‐367. doi:10.1002/jrsm.1547 35088937

