# Supplementary material for: The prevalence of apathy in Lewy body dementia: A systematic review and meta‐analysis
Source: Alzheimers Dement. 2025 Jul 3;21(7):e70425. doi: 10.1002/alz.70425 (PMC12226429; doi:10.1002/alz.70425)
Supplement: Supplementary file 1 — Supporting Information [file ALZ-21-e70425-s003.pdf]

Supplementary figures: Sensitivity analyses

Sensitivity analysis: removing studies with overlapping cohorts (Supplementary figures 1 – 5)

Supplementary figure 1. Average pooled prevalence of apathy in LBD

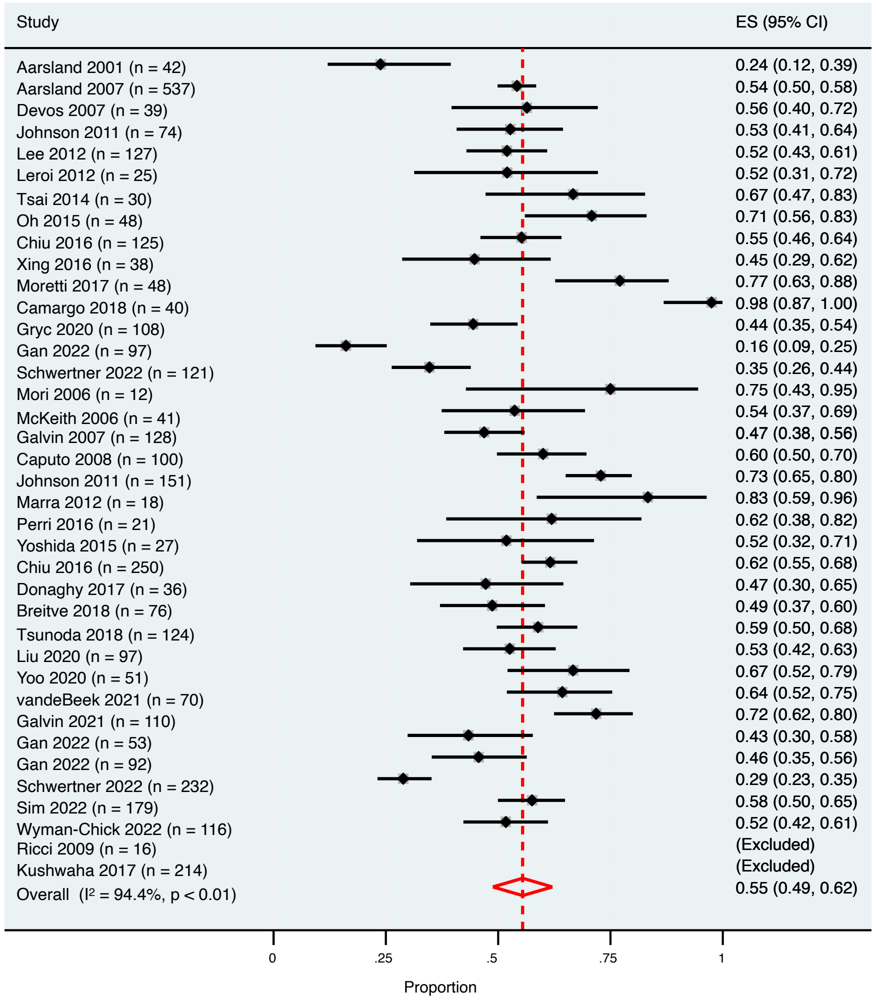

Supplementary figure 2. Average pooled prevalence of apathy in DLB

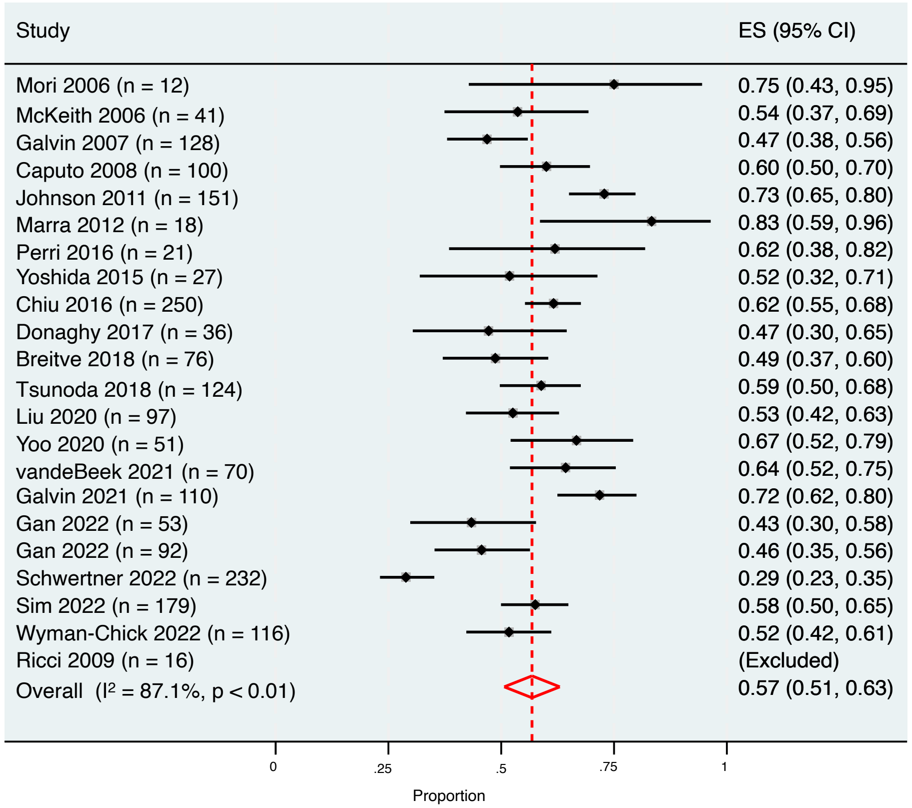

Supplementary figure 3. Average pooled prevalence of apathy in PDD

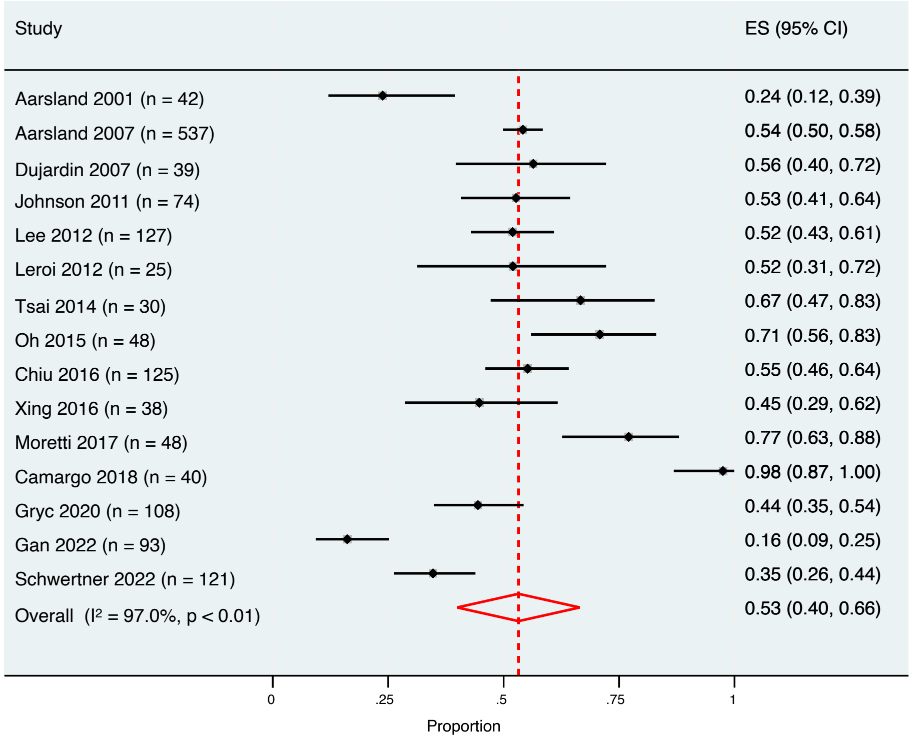

Supplementary figure 4. Average pooled prevalence of apathy in combined MCI (LB- and PD-MCI)

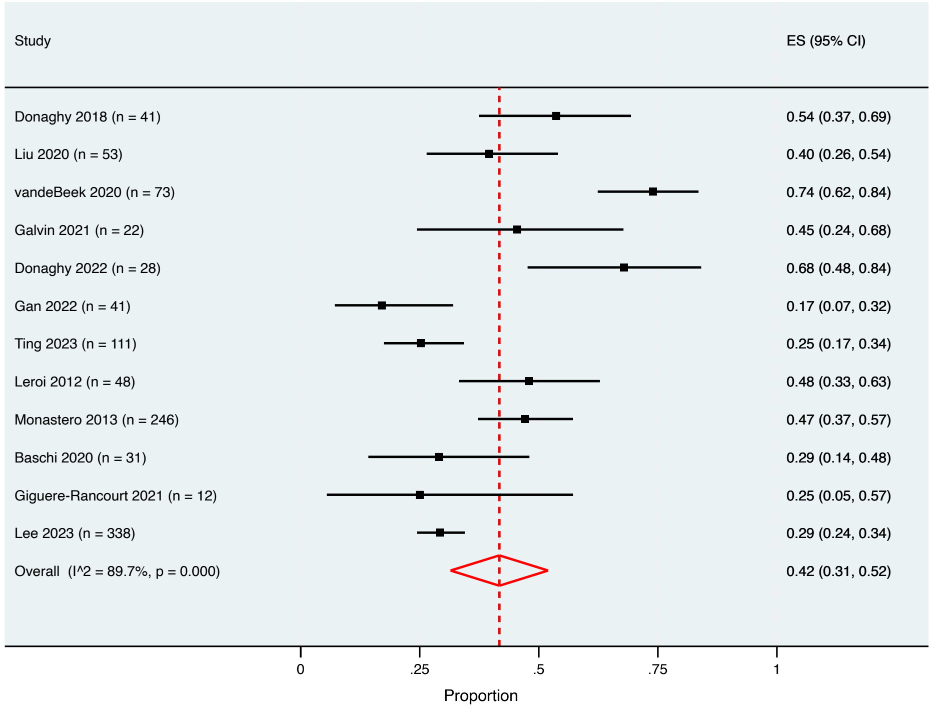

Supplementary figure 5. Average pooled prevalence of apathy in LB-MCI

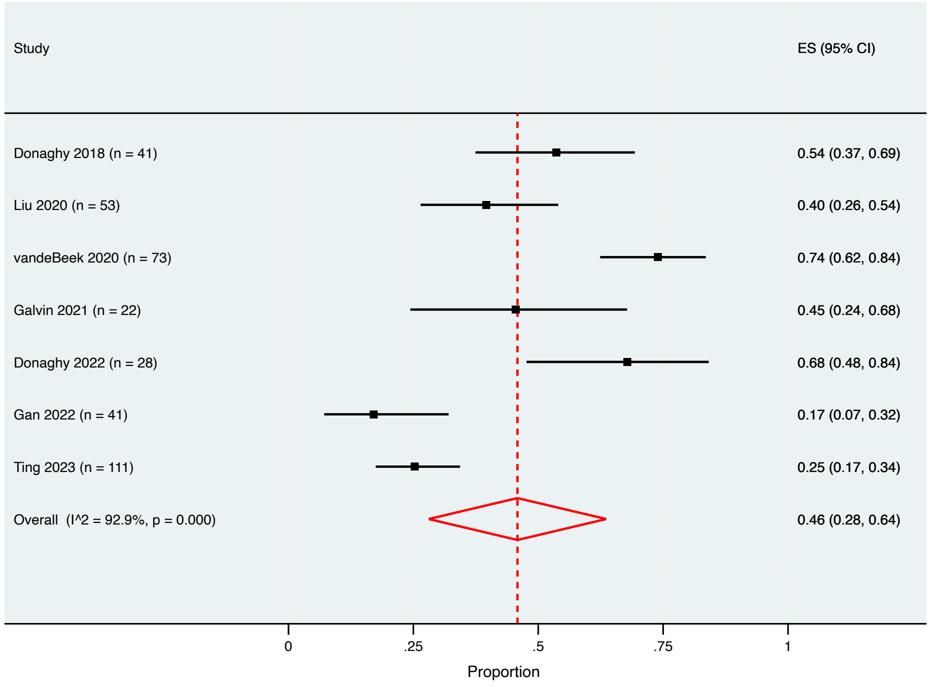

Sensitivity analysis: studies with cohorts of more than 50 (Supplementary figures 6 – 9)

Supplementary figure 6. Average pooled prevalence of apathy in LBD

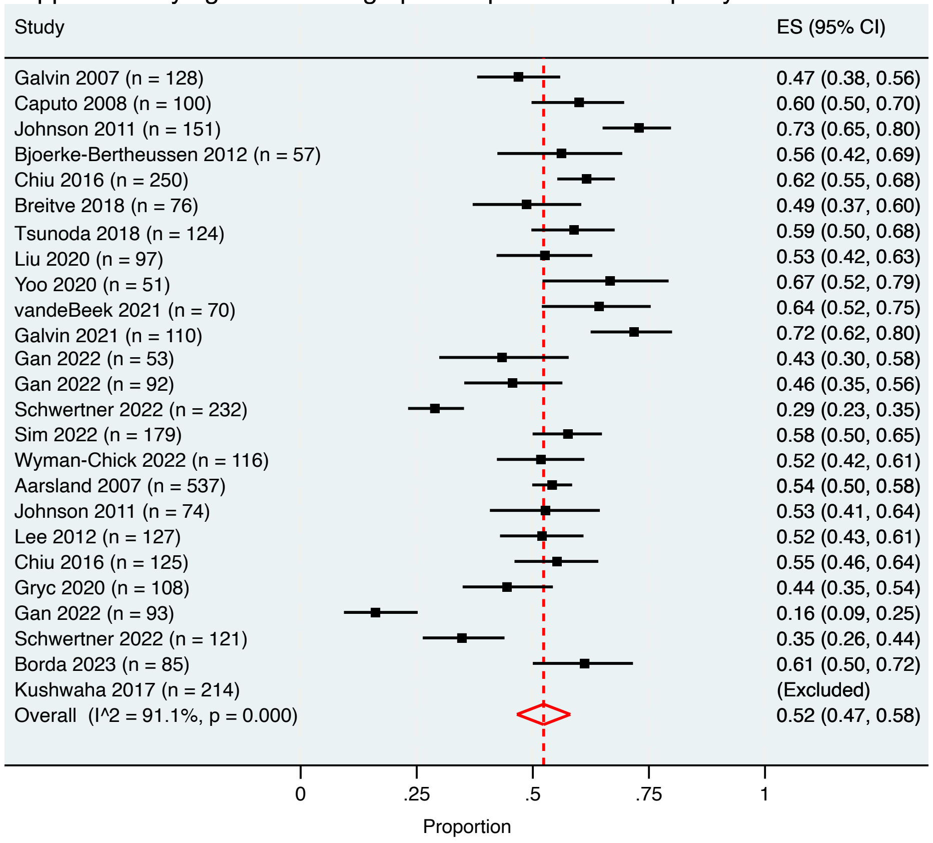

Supplementary figure 7. Average pooled prevalence of apathy in DLB

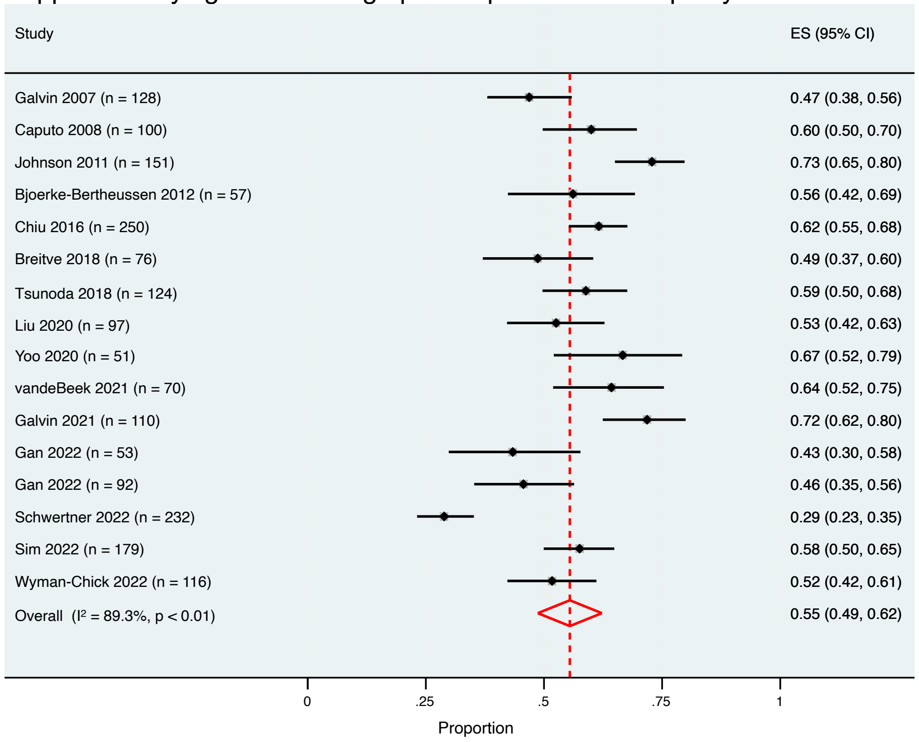

Supplementary figure 8. Average pooled prevalence of apathy in PDD

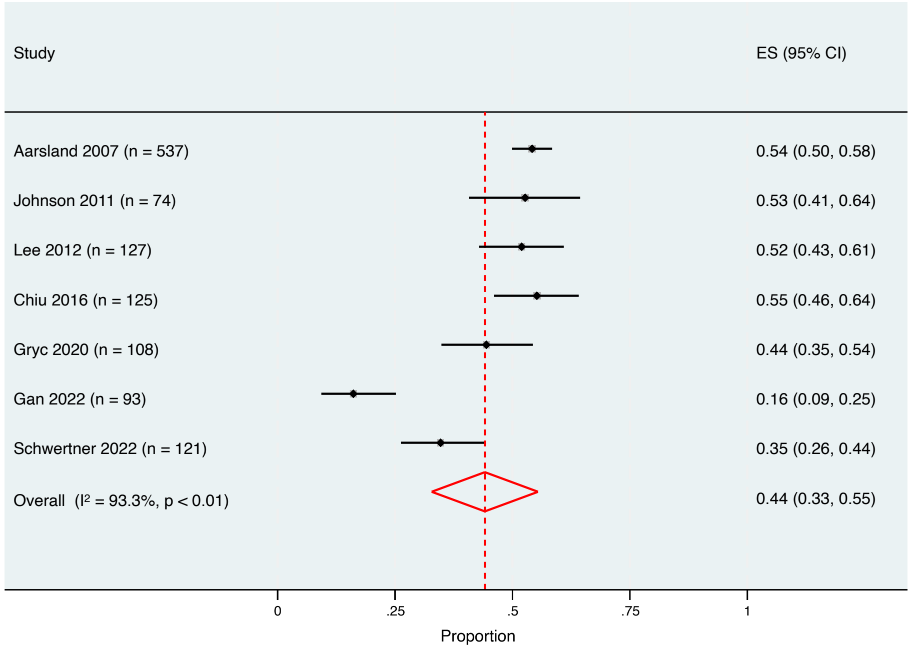

Supplementary figure 9. Average pooled prevalence of apathy in combined MCI (LB- and PD-MCI)

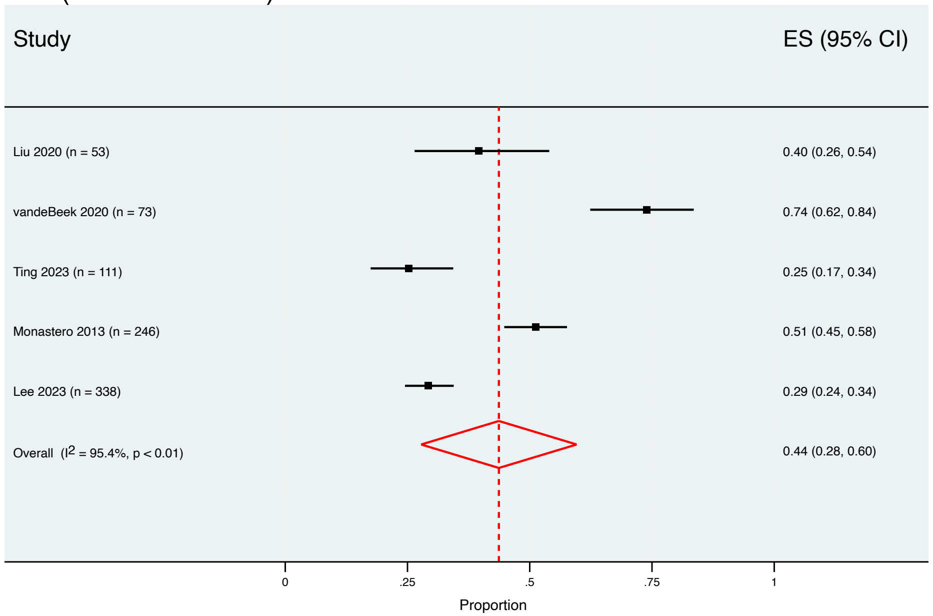

Sensitivity analysis: studies utilising the NPI to diagnose apathy (Supplementary figure10 – 14)

Supplementary figure 10. Average pooled prevalence of apathy in LBD

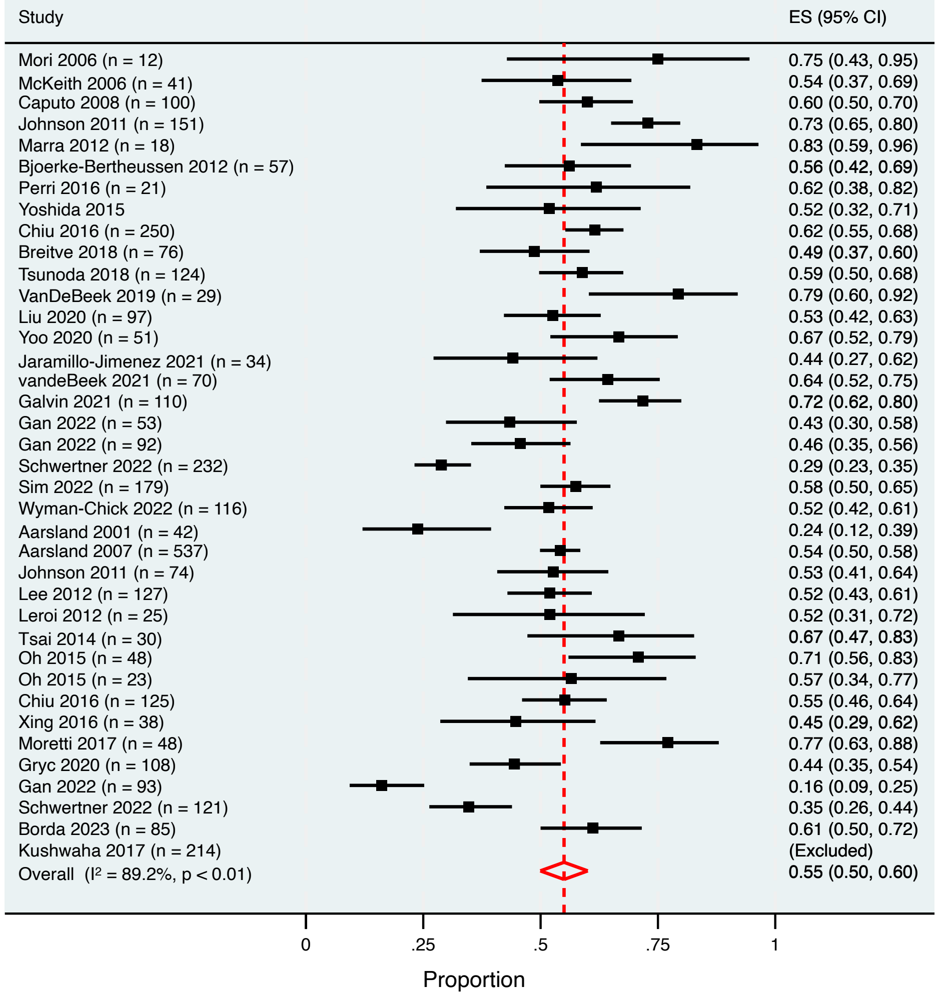

Supplementary figure 11. Average pooled prevalence of apathy in DLB

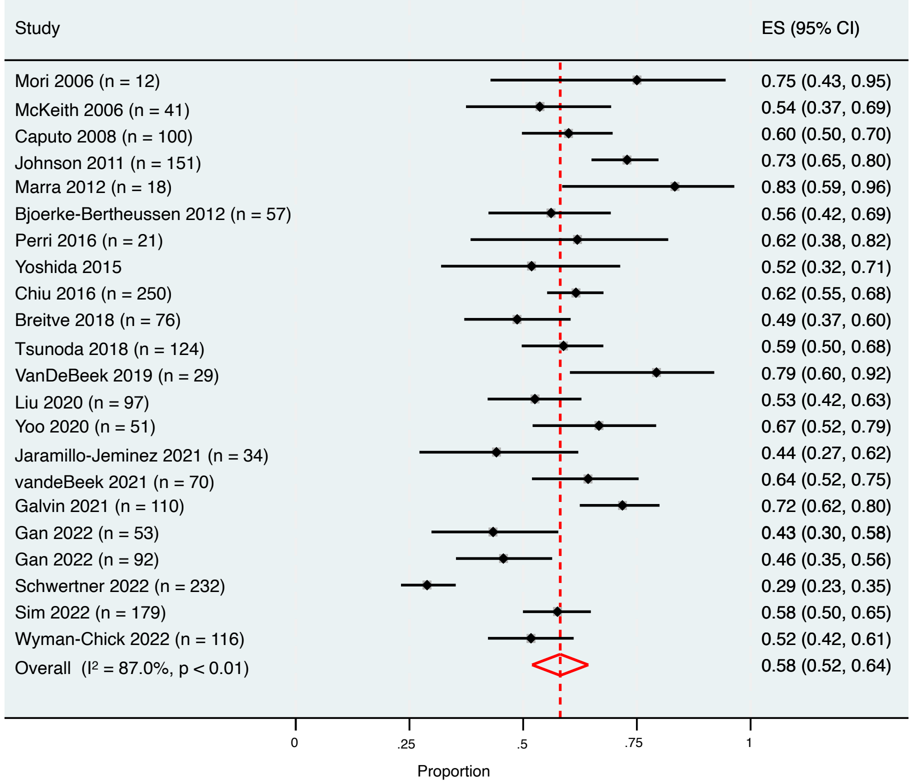

Supplementary figure 12. Average pooled prevalence of apathy in PDD

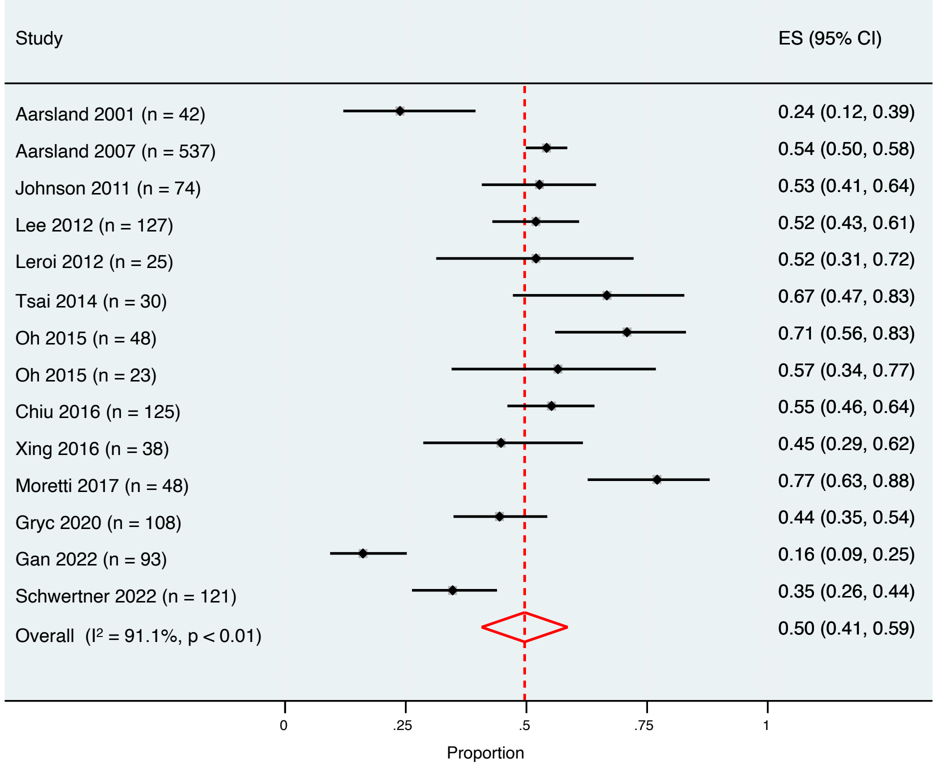

Supplementary figure 13. Average pooled prevalence of apathy in combined MCI

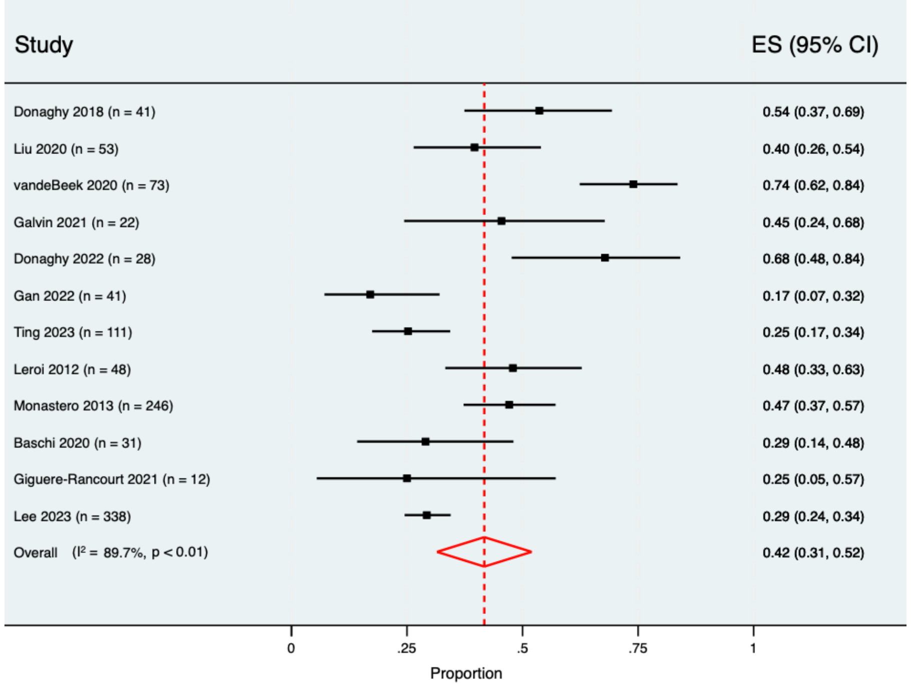

Supplementary figure 14. Average pooled prevalence of apathy in LB-MCI

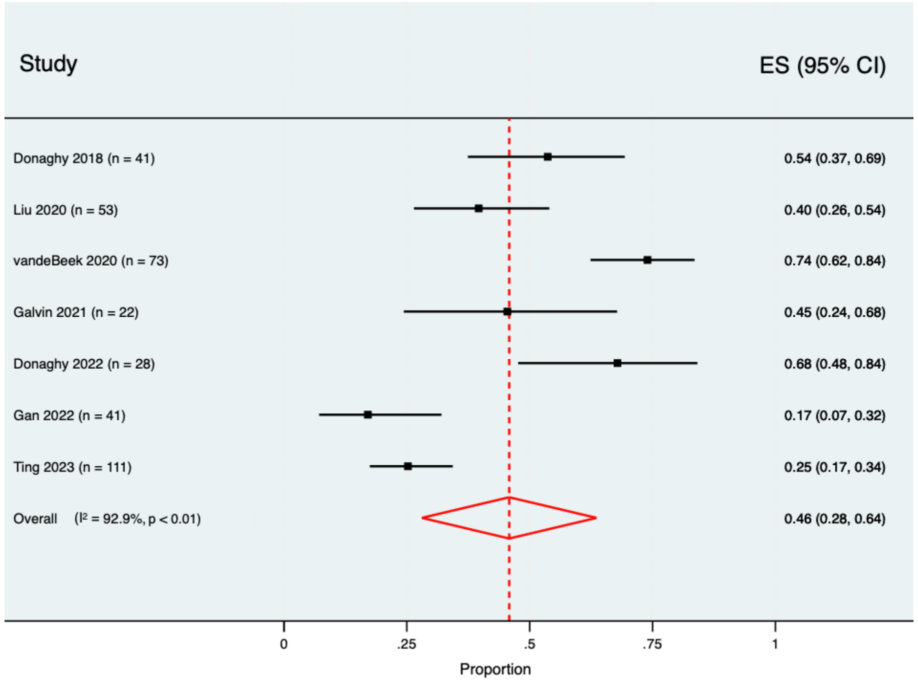

Supplementary figures 1 – 14: CI = confidence interval, ES = effect size
