# Supplementary material for: The prevalence of apathy in Lewy body dementia: A systematic review and meta‐analysis
Source: Alzheimers Dement. 2025 Jul 3;21(7):e70425. doi: 10.1002/alz.70425 (PMC12226429; doi:10.1002/alz.70425)
Supplement: Supplementary file 2 — Supporting Information [file ALZ-21-e70425-s001.docx]

Appendix A. Search strategies for Ovid, Web of Science and CINAHL platforms.

*Ovid (Embase, Medline, PsycInfo)*

| 1 | *diffuse Lewy body disease/ or (DLB or LBD).mp. or *Lewy body/ or lewy body.mp. |
| --- | --- |
| 2 | *Parkinson disease/ or (parkinson disease or parkinsons disease or parkinson's disease).mp. AND (*dementia/ or dementia.mp. or cognitive decline.mp. or *mild cognitive impairment/ or mci.mp.) |
| 3 | 1 or 2 |
| 4 | ((behavio* and psychological symptoms of dementia) or BPSD or neuropsychiatric symptoms or neuro-psychiatric symptoms or psycho-behavio* symptoms or behavio* symptoms or psychiatric symptoms or psychological symptoms or non-cognitive symptoms).mp. |
| 5 | *apathy/ or (indifference or amotivation).mp. or avolition.mp. |
| 6 | *Neuropsychiatric inventory/ |
| 7 | 3 and (4 or 5 or 6) |

*Web of Science*

| 1 | ALL=(lewy body or DLB or LBD) |
| --- | --- |
| 2 | (ALL=("parkinson disease" OR "parkinsons disease" OR "parkinson's disease")) AND ALL=(dementia or "cognitive decline" OR "mild cognitive impairment" OR MCI) |
| 3 | (#2) OR #1 |
| 4 | ALL=(((behavio* AND "psychological symptoms of dementia") OR BPSD OR "neuropsychiatric symptoms" OR "neuro-psychiatric symptoms" OR "psycho-behavio* symptoms" OR "behavio* symptoms" OR "psychiatric symptoms" OR "psychological symptoms" OR "non-cognitive symptoms")) |
| 5 | ALL=(apathy OR (indifference OR amotivation) OR avolition) |
| 6 | ALL=("neuropsychiatric inventory") |
| 7 | ((#4) OR #5) OR #6 |
| 8 | **(#7) AND #3** |

*CINAHL*

| S1 | ( (MM "diffuse Lewy body disease") OR (DLB OR LBD) OR (MM "Lewy body") OR "lewy body" ) OR ( (MM "Parkinson disease") OR ("parkinson disease" OR "parkinsons disease" OR "parkinson's disease") AND (MM dementia) OR dementia OR "cognitive decline") |
| --- | --- |
| S2 | ((behavio* AND "psychological symptoms of dementia") OR BPSD OR "neuropsychiatric symptoms" OR "neuro-psychiatric symptoms" OR "psycho-behavio* symptoms" OR "behavio* symptoms" OR "psychiatric symptoms" OR "psychological symptoms" OR "non-cognitive symptoms") |
| S3 | (MM apathy) OR (indifference OR amotivation) OR avolition |
| S4 | (MM "Neuropsychiatric inventory") |
| S5 | S2 OR S3 OR S4 |

Appendix B. Study quality assessment (JBI Checklist for Prevalence Studies)

|  | Quality assessment questions (rater 1 response – not shaded, rater 2 response – shaded) | | | | | | | | | | | | | | | | | |
| --- | --- | --- | --- | --- | --- | --- | --- | --- | --- | --- | --- | --- | --- | --- | --- | --- | --- | --- |
| Study | Q1 | Q1 | Q2 | Q2 | Q3 | Q3 | Q4 | Q4 | Q5 | Q5 | Q6 | Q6 | Q7 | Q7 | Q8 | Q8 | Q9 | Q9 |
| Aarsland 2001 | Yes | Yes | Unc | Yes | N/A | N/A | Yes | Yes | Yes | Yes | Yes | Yes | Yes | Yes | N/A | N/A | N/A | N/A |
| Aarsland 2007 | No | No | Unc | Yes | N/A | N/A | Yes | Yes | Yes | Yes | Yes | Yes | Yes | Yes | N/A | N/A | N/A | N/A |
| Baschi 2020 | No | No | Yes | Yes | N/A | N/A | Yes | Yes | Yes | Yes | Yes | Yes | Yes | Yes | N/A | N/A | N/A | N/A |
| Bjoerke-Bertheussen 2012 | Yes | Yes | Yes | Yes | N/A | N/A | Yes | Yes | Unc | Yes | Yes | Yes | Yes | Yes | N/A | N/A | N/A | N/A |
| Borda 2023 | Yes | Yes | Yes | Yes | N/A | N/A | Yes | Yes | Yes | Yes | Yes | Yes | Yes | Yes | N/A | N/A | N/A | N/A |
| Breitve 2018 | Yes | Yes | Unc | Unc | N/A | N/A | Yes | Yes | Yes | Yes | Yes | Yes | Yes | Yes | N/A | N/A | N/A | N/A |
| Camargo 2016 | Yes | Yes | Unc | Yes | N/A | N/A | Yes | Yes | Yes | Yes | Yes | Yes | Yes | Yes | N/A | N/A | N/A | N/A |
| Camargo 2018 | Yes | Yes | Unc | Yes | N/A | N/A | Yes | Yes | Yes | Yes | Yes | Yes | Yes | Yes | N/A | N/A | N/A | N/A |
| Caputo 2008 | Yes | Yes | Yes | Yes | N/A | N/A | Yes | Yes | Yes | Yes | Yes | Yes | Yes | Yes | N/A | N/A | N/A | N/A |
| Chiu 2016 | Yes | Yes | Yes | Yes | N/A | N/A | Yes | Yes | Unc | Yes | Yes | Yes | Yes | Yes | N/A | N/A | N/A | N/A |
| Dujardin 2007 | Unc | Unc | Unc | Unc | N/A | N/A | Yes | Yes | Yes | Yes | Yes | Yes | Yes | Yes | N/A | N/A | N/A | N/A |
| Donaghy 2017 | Yes | Yes | Unc | Unc | N/A | N/A | Yes | Yes | Unc | Unc | Yes | Yes | Yes | Yes | N/A | N/A | N/A | N/A |
| Donaghy 2018 | Yes | Yes | Unc | Unc | N/A | N/A | Yes | Yes | Yes | Yes | Yes | Yes | Yes | Yes | N/A | N/A | N/A | N/A |
| Donaghy 2022 | Yes | Yes | Unc | Unc | N/A | N/A | Yes | Yes | Yes | Yes | Yes | Yes | Yes | Yes | N/A | N/A | N/A | N/A |
| Galvin 2021 | Yes | Yes | Unc | Unc | N/A | N/A | Yes | Yes | Yes | Yes | Yes | Yes | Yes | Yes | N/A | N/A | N/A | N/A |
| Galvin 2007 | Yes | Yes | Unc | No | N/A | N/A | Yes | Yes | Yes | Yes | Yes | Yes | Yes | Yes | N/A | N/A | N/A | N/A |
| Gan 2022 | Yes | Yes | Yes | Yes | N/A | N/A | Yes | Yes | Unc | Unc | Yes | Yes | Yes | Yes | N/A | N/A | N/A | N/A |
| Gan 2022 | Yes | Yes | Unc | Unc | N/A | N/A | Yes | Yes | Yes | Yes | Yes | Yes | Yes | Yes | N/A | N/A | N/A | N/A |
| Giguere-Rancourt 2021 | Yes | Yes | Unc | Yes | N/A | N/A | Yes | Yes | Yes | Yes | Yes | Yes | Yes | Yes | N/A | N/A | N/A | N/A |
| Gryc 2020 | Yes | Yes | Unc | Yes | N/A | N/A | Yes | Yes | Yes | Yes | Yes | Yes | Yes | Yes | N/A | N/A | N/A | N/A |
| Jaramillo-Jimenez 2021 | No | No | Yes | Yes | N/A | N/A | Yes | Yes | Yes | Yes | Yes | Yes | Yes | Yes | N/A | N/A | N/A | N/A |
| Johnson 2011 | Yes | Yes | Yes | Yes | N/A | N/A | Yes | Yes | Yes | Yes | Yes | Yes | Yes | Yes | N/A | N/A | N/A | N/A |
| Kushwaha 2017 | Yes | Yes | Yes | Yes | N/A | N/A | Yes | Yes | Yes | Yes | Yes | Yes | Yes | Yes | N/A | N/A | N/A | N/A |
| Lee 2012 | Yes | Yes | Unc | Unc | N/A | N/A | Yes | Yes | Yes | Yes | Yes | Yes | Yes | Yes | N/A | N/A | N/A | N/A |
| Lee 2023 | Yes | Yes | Yes | Yes | N/A | N/A | Yes | Yes | Yes | Yes | Yes | Yes | Yes | Yes | N/A | N/A | N/A | N/A |
| Leroi 2012 | No | Unc | Yes | Yes | N/A | N/A | Yes | Yes | Yes | Yes | Yes | Yes | Yes | Yes | N/A | N/A | N/A | N/A |
| Liu 2020 | Yes | Yes | Yes | Yes | N/A | N/A | Yes | Yes | Yes | Yes | Yes | Yes | Yes | Yes | N/A | N/A | N/A | N/A |
| Marra 2012 | Unc | Unc | Unc | Unc | N/A | N/A | Yes | Yes | Yes | Yes | Yes | Yes | Yes | Yes | N/A | N/A | N/A | N/A |
| McKeith 2006 | Yes | Yes | Yes | Yes | N/A | N/A | Yes | Yes | Unc | Unc | Yes | Yes | Yes | Yes | N/A | N/A | N/A | N/A |
| Monastero 2013 | Yes | Yes | Yes | Yes | N/A | N/A | Yes | Yes | Yes | Yes | Yes | Yes | Yes | Yes | N/A | N/A | N/A | N/A |
| Moretti 2017 | No | No | Unc | Unc | N/A | N/A | Yes | Yes | Yes | Unc | Yes | Yes | Yes | Yes | N/A | N/A | N/A | N/A |
| Mori 2006 | Yes | Yes | Unc | Unc | N/A | N/A | Yes | Yes | Yes | Yes | Yes | Yes | Yes | Yes | N/A | N/A | N/A | N/A |
| Oh 2015 | No | No | Unc | Unc | N/A | N/A | Yes | Yes | Unc | Unc | Yes | Yes | Yes | Yes | N/A | N/A | N/A | N/A |
| Oh 2015 | Yes | Yes | Unc | Unc | N/A | N/A | Yes | Yes | Yes | Yes | Yes | Yes | Yes | Yes | N/A | N/A | N/A | N/A |
| Perri 2016 | Yes | Yes | Yes | Yes | N/A | N/A | Yes | Yes | Unc | Unc | Yes | Yes | Yes | Yes | N/A | N/A | N/A | N/A |
| Ricci 20019 | Yes | Yes | Yes | Yes | N/A | N/A | No | Yes | Yes | Yes | Yes | Yes | Yes | Yes | N/A | N/A | N/A | N/A |
| Schwertner 2022 | Yes | Yes | Unc | Yes | N/A | N/A | Yes | Yes | Yes | Yes | Yes | Yes | Yes | Yes | N/A | N/A | N/A | N/A |
| Siepel 2016 | Yes | Yes | No | Yes | N/A | N/A | Yes | Yes | Unc | Yes | Yes | Yes | Yes | Yes | N/A | N/A | N/A | N/A |
| Sim 2022 | Yes | Yes | Unc | Yes | N/A | N/A | Yes | Yes | Yes | Yes | Yes | Yes | Yes | Yes | N/A | N/A | N/A | N/A |
| Ting 2023 | Yes | Yes | Yes | Yes | N/A | N/A | Yes | Yes | Yes | Yes | Yes | Yes | Yes | Yes | N/A | N/A | N/A | N/A |
| Tsai 2014 | Unc | Unc | Unc | Unc | N/A | N/A | Yes | Yes | Yes | Yes | Yes | Yes | Yes | Yes | N/A | N/A | N/A | N/A |
| Tsunado 2018 | Yes | Yes | Yes | Yes | N/A | N/A | Yes | Yes | Yes | Yes | Yes | Yes | Yes | Yes | N/A | N/A | N/A | N/A |
| VanDeBeek 2019 | Yes | Yes | Unc | Yes | N/A | N/A | Yes | Yes | Unc | Unc | Yes | Yes | Yes | Yes | N/A | N/A | N/A | N/A |
| VanDeBeek 2020 | Yes | Yes | Unc | Unc | N/A | N/A | Yes | Yes | No | No | Yes | Yes | Yes | Yes | N/A | N/A | N/A | N/A |
| VanDeBeek 2021 | Yes | Yes | No | Yes | N/A | N/A | Yes | Yes | No | No | Yes | Yes | Yes | Yes | N/A | N/A | N/A | N/A |
| Wyman-Chick 2022 | Yes | Yes | Unc | Yes | N/A | N/A | Yes | Yes | Yes | Yes | Yes | Yes | Yes | Yes | N/A | N/A | N/A | N/A |
| Xing 2016 | Yes | Yes | Unc | Unc | N/A | N/A | Yes | Yes | Yes | Yes | Yes | Yes | Yes | Yes | N/A | N/A | N/A | N/A |
| Yoo 2020 | Yes | Yes | Yes | Yes | N/A | N/A | Yes | Yes | Yes | Yes | Yes | Yes | Yes | Yes | N/A | N/A | N/A | N/A |
| Yoshida 2015 | Yes | Yes | Unc | Unc | N/A | N/A | Yes | Yes | Yes | Yes | Yes | Yes | Yes | Yes | N/A | N/A | N/A | N/A |

Q1. Was the sample frame appropriate to address the target population?

Q2. Were study participants sampled in an appropriate way?

Q3. Was the sample size adequate?

Q4. Were the study subjects and the setting described in detail?

Q5. Was the data analysis conducted with sufficient coverage of the identified sample?

Q6. Were valid methods used for the identification of the condition?

Q7. Was the condition measured in a standard, reliable way for all participants?

Q8. Was there appropriate statistical analysis?

Q9. Was the response rate adequate, and if not, was the low response rate managed appropriately?

N/A = not applicable; unc = unclear, as there was not enough description in the article to answer this question
